# Comparative study on secondary metabolites from different citrus varieties in the production area of Zhejiang

**DOI:** 10.3389/fnut.2023.1159676

**Published:** 2023-05-11

**Authors:** Mei Lin, Chengnan Xu, Xueying Gao, Weiqing Zhang, Zhoulin Yao, Tianyu Wang, Xianju Feng, Yue Wang

**Affiliations:** ^1^Zhejiang Citrus Research Institute, Taizhou, China; ^2^Center for Reproductive Medicine, Ren Ji Hospital, School of Medicine, Shanghai Jiao Tong University, Shanghai, China; ^3^Shanghai Key Laboratory for Assisted Reproduction and Reproductive Genetics, Shanghai, China

**Keywords:** citrus pulp, citrus peel, secondary metabolites, distribution pattern, PCA

## Abstract

To investigate the distribution pattern of bioactive components and their correlations between citrus varieties, we thoroughly analyzed secondary metabolites (including flavonoids, phenolic acids, carotenoids, and limonoids) in the peel and pulp of 11 citrus varieties from the production area of Zhejiang. Citrus peels accumulated metabolites far more than the pulp, and the accumulation varied significantly between species. Flavonoids were the most abundant compounds, followed by phenolic acids, with carotenoids and limonoids being far less abundant than the first two, but limonoids were more abundant than carotenoids. Hesperidin was the main flavonoid in most varieties, but cocktail grapefruit and Changshanhuyou contained naringin, with Ponkan having the most abundant polymethoxylated flavones (PMFs). The major components of phenolic acids, carotenoids, and limonoids were ferulic acid, β-cryptoxanthin, and limonin, respectively. Principal component analysis (PCA) and hierarchical cluster analysis (HCA) indicated that these components were mostly correlated with each other, and these citrus varieties could be categorized into four groups by pulp and three groups by peel. The obtained results filled the data gap for secondary metabolites from local citrus and could provide data references for citrus resource utilization, selection and breeding of superior varieties, and other research.

## 1. Introduction

Citrus fruits are rich in nutrients such as sugars, acids, vitamins, and minerals, as well as bioactive components, including flavonoids, phenolic acids, carotenoids, and limonoids (1–4). These secondary metabolites are beneficial to human health, including participating in human metabolism, regulating physiological activities, scavenging free radicals in the body, and having anti-bacterial and anti-cancer properties (5–9). Therefore, citrus fruits are popular and are the most abundant fruits grown and consumed worldwide (10). China is the world's largest producer and consumer of citrus, with the largest production and planted area in the world (11). However, at the same time, there are problems such as single cultivars, a concentrated marketing period, and a lagging processing industry in each production area. For this reason, in recent years, the production area of Zhejiang has adjusted its citrus cultivation structure by vigorously developing early-ripening and late-ripening hybrid citrus varieties.

Hybrid citrus varieties have some combined characteristics of orange, tangerine, and grapefruit with good flavor and high nutritional value, but the performance of the introduced citrus varied in different areas. At the same time, current research on these introduced citruses is more focused on their biological performance, cultivation technology, and so on, while comprehensive research on the nutritional and functional components is lacking. The polyphenols in citrus are influenced by multiple factors, variety, in particular, and climate, environment, and ripening period, in general. Therefore, numerous studies on citrus varieties from different regions could provide unique resources for research on the biological activity of citrus fruits (12, 13). Currently, studies on active ingredients have been mainly focused on one type of functional components, such as the phenolic content and distribution of citrus fruits (14), the phenolic and antioxidant activity of citrus peel (11, 15), and carotenoids (16, 17) and limonoids (18) of citrus fruits. However, few studies have been reported on the comprehensive studies of these abovementioned functional components. Thus, a full investigation of multiple functional components in 11 citrus varieties in the production area of Zhejiang could facilitate the utilization, selection, and breeding of citrus resources. In this study, we aimed to thoroughly analyze secondary metabolites, including flavonoids, phenolic acids, carotenoids, and limonoids, in the peel and pulp of local citrus varieties to investigate their distribution pattern and their correlation between varieties.

## 2. Materials and methods

### 2.1. Materials and reagents

A total of 11 citrus varieties (brief information on citrus varieties can be seen in Supplementary Table 1) were collected from the germplasm nursery of the Zhejiang Citrus Research Institute, and all trees were cultivated and managed under the same conditions. The geographic coordinates of the sampling site were listed as 28°38'36“ N and 121°9'30” E, with an altitude of 50 m. All fruits were harvested at their commercial maturity stage (from October 2021 to January 2022), based on size uniformity and external color. For each variety, 6 trees with similar growth were selected, and 6 fruits per plant were randomly picked in all directions. In total, 36 fruits were divided into 3 groups as a repetition.

Then, the surface of the fruits was gently wiped with a gauze. After that, each fruit was cross-cut into four parts, and one piece of the diagonal parts was selected to manually separate the peel and pulp (the pulp retained the capsule but removed the seeds). Then, the above peel and pulp were ground in liquid nitrogen and stored at −20°C for later use.

Eriocitrin, neoeriocitrin, narirutin, neohesperidin, rhoifolin, vanillin, poncirin, naringenin, hesperetin, apigenin, sinensetin, nobiletin, and tangeretin, 13 standard compounds with purity greater than 95%, and other parts of standard compounds, such as lutein (≥ 97%), zeaxanthin (≥ 95%), β-cryptoxanthin (≥ 97%), α-carotene (≥ 98%), and β-carotene (≥ 97%), were purchased from Sigma-Aldrich (St Louis, MO, USA). Hesperidin (> 98.0%) and naringin (> 99.0%) were purchased from Dr. Ehrenstorfer GmbH (Augsburg, Germany). Vanillic acid (≥ 97%), caffeic acid (≥ 98%), protocatechuic acid (≥ 98%), phydroxybenzonic acid (≥ 99%), *p*-coumaric acid (≥ 97%), ferulic acid (≥ 97%), sinapic acid (≥ 98%), limonin (≥ 98%), and nomilin (≥ 98%) were purchased from Shanghai Yuanye Bio-Technology Co., Ltd. (Shanghai, China). All organic reagents at HPLC grade and other reagents at analytical grade were used in the experiments. Besides, deionized water was obtained from a Milli-Q water purification system (Millipore, Billerica, USA).

### 2.2. Sample preparation

The extraction process was performed according to previous literature (11, 16, 19, 20). Briefly, to extract flavonoids and limonoids, the citrus tissues were sonicated with 10 ml of methanol-dimethyl sulfoxide solution (*v*/*v*=1:1) for 30 min and centrifuged at 10,000 rpm for 5 min. The abovementioned procedure was repeated three times, and the supernatants were collected. The final volume was made up to 50 ml for peel samples (25 ml for pulp). To extract phenolic acids, the citrus tissues were vortexed with 10 ml of NaOH solution (4 mol/L) for 4 h, and then the pH of the solution was adjusted to 2.0 and centrifuged. The supernatants were extracted with ethyl acetate-methyl tert-butyl ether (MTBC) solution (*v*/*v*=1:1) three times. The extracts were combined and concentrated to dryness by rotary evaporation, and the volume was fixed at 5 ml with a 50% methanol aqueous solution. For carotenoid extraction, the citrus tissues were mixed with the hexane-acetone-ethanol solution (*v*/*v*/*v*=2:1:1) for 8 min of high-speed homogenization and centrifugation, and then, the collected organic phase was concentrated to dryness by rotary evaporation and redissolved in MTBC solution. Next, the above solution was mixed with KOH-methanol solution (*v*/*v*=1:10) for saponification in the dark for 12 h. Then, the saponification solution was extracted two times by the MTBC solution, and the organic phase was collected. Finally, the organic phase was dried with nitrogen and redissolved in an MTBC solution. Before UPLC analysis, all extract solutions were filtered through a 0.22-μm organic membrane.

### 2.3. UPLC analysis of flavonoids

An ACQUITY UPLC system with Empower version 4.1 software was applied to quantify flavonoids in extracts according to previous methods (21). The system comprises a 2,996 PDA detector (Waters, Milford, MA, USA) and an ACQUITY UPLC BEH C_18_ column for separation (2.1 mm × 100 mm and 1.7 μm particle size), among others. At the column temperature of 35°C, 3 μl of the sample was injected into the system. The mobile phase consists of 0.2% acetic acid aqueous solution (A) and 100% methanol (B), with a gradient elution as follows: The flow rate was set at 0.3 ml/min, with a 10% B linear gradient increasing to 30% in the first 5 min, followed by another linear gradient increasing to 80% in the next 5 min, then stabilizing at 80% for 5 min, ending with a linear gradient back to 10% in 1 min. PMFs were monitored at 330 nm, while other flavonoids were detected at 283 nm. The flavonoids were qualitatively analyzed depending on their retention time and characteristic spectrum; furthermore, an external standard method was used for quantification.

### 2.4. UPLC analysis of phenolic acids

Phenolic acids were quantified in extracts using the abovementioned UPLC system according to previous reports (11), with constant injection volume, column temperature, and flow rate. However, the mobile phase changed to 3% formic acid aqueous solution (A) and 100% methanol (B). The mobile phase began with 95% A and a linear gradient down to 75% in the first 5 min, followed by an isocratic step of 75% for 3 min, then a linear gradient descent to 20% in the next 4 min, and finally linear gradient increase to 95% in 4 min. Protocatechuic acid, phydroxybenzonic acid, and vanillic acid were detected at 220 nm, whereas 320 nm was set for caffeic acid, *p*-coumaric acid, ferulic acid, and sinapic acid.

### 2.5. UPLC analysis of carotenoids

The same apparatus and a C_30_ column (4.6 mm × 100 mm and 3 μm particle size) were utilized for carotenoid determination, according to previous literature (16). The mobile phase A was *v*_(acetonitrile)_:*v*_(methanol)_:*v*_(water)_ =81:14:5, and the mobile phase B was *v*_(methanol)_:*v*_(ethyl acetate)_=68:32; moreover, both phases A and B included 0.05% triethylamine. The gradient elution process was as follows: the flow rate was 0.5 ml/min and kept at 100% A for the first 0.3 min, followed by a linear gradient descent to 0% A and holding for 8 min, followed by a linear gradient back to 100% A in 2 min and stable at 100% A for 3 min to end. For the whole experiment, 3 μl of the sample was injected, with the column temperature set at 35°C and the detection wavelength set at 450 nm. The carotenoids were judged by their retention time and quantified by the standard curve of the standard compound (*R*^2^ ≥ 0.9990).

### 2.6. UPLC analysis of limonoids

The apparatus and experimental parameters for limonoid determination were the same as for flavonoid analysis, and the mobile phase was 40% acetonitrile in water with an isocratic elution for 8 min (22). Besides, the detection wavelength was set at 210 nm.

### 2.7. Statistics analysis

All citrus samples were sorted by peel and pulp and were prepared and analyzed in triplicates. Data statistics were performed using the SPSS 24.0 software (IBM SPSS, Armonk, USA), and all results were expressed as the means ± standard deviation (SD). The standard error was calculated, while multiple comparisons were undertaken by analysis of variance (ANOVA) and Duncan's new multiple range test (*p* < 0.05). PCA was performed among 29 metabolites and 11 varieties in fruit flesh by the R package “FactoMineR (version 2.4)”. All the metabolites' contents were scaled before the analysis. Heatmaps were generated by the R package “pheatmap (version 1.0.12)”. The hierarchical cluster was analyzed *via* the “hclust” function by the R package (version 4.0.3) with the agglomeration methods of “ward.D2” according to the Euclidean distance.

## 3. Results

**Figure 1:**
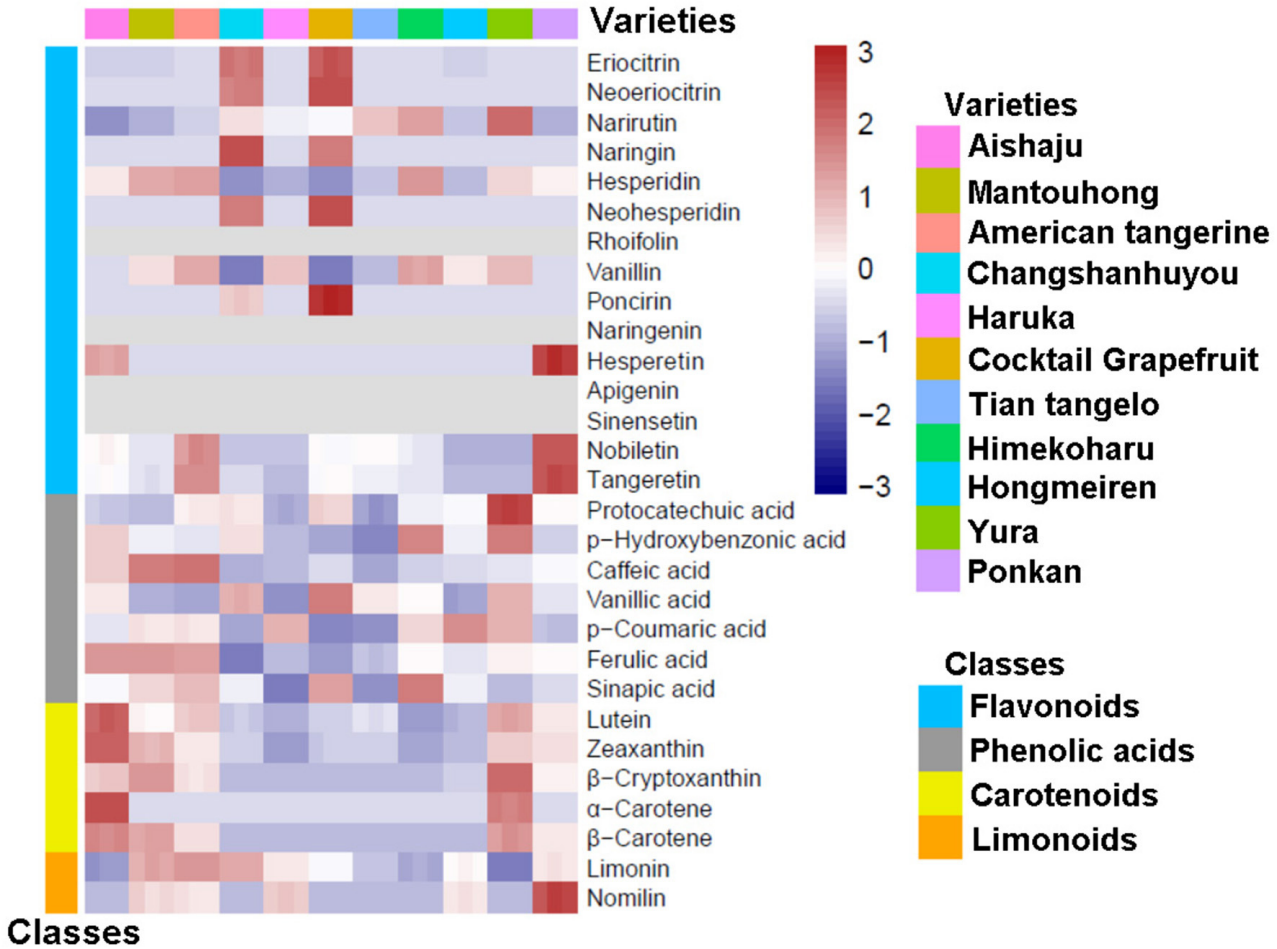
A heatmap of secondary metabolites in pulp from different citrus varieties.

**Figure 2:**
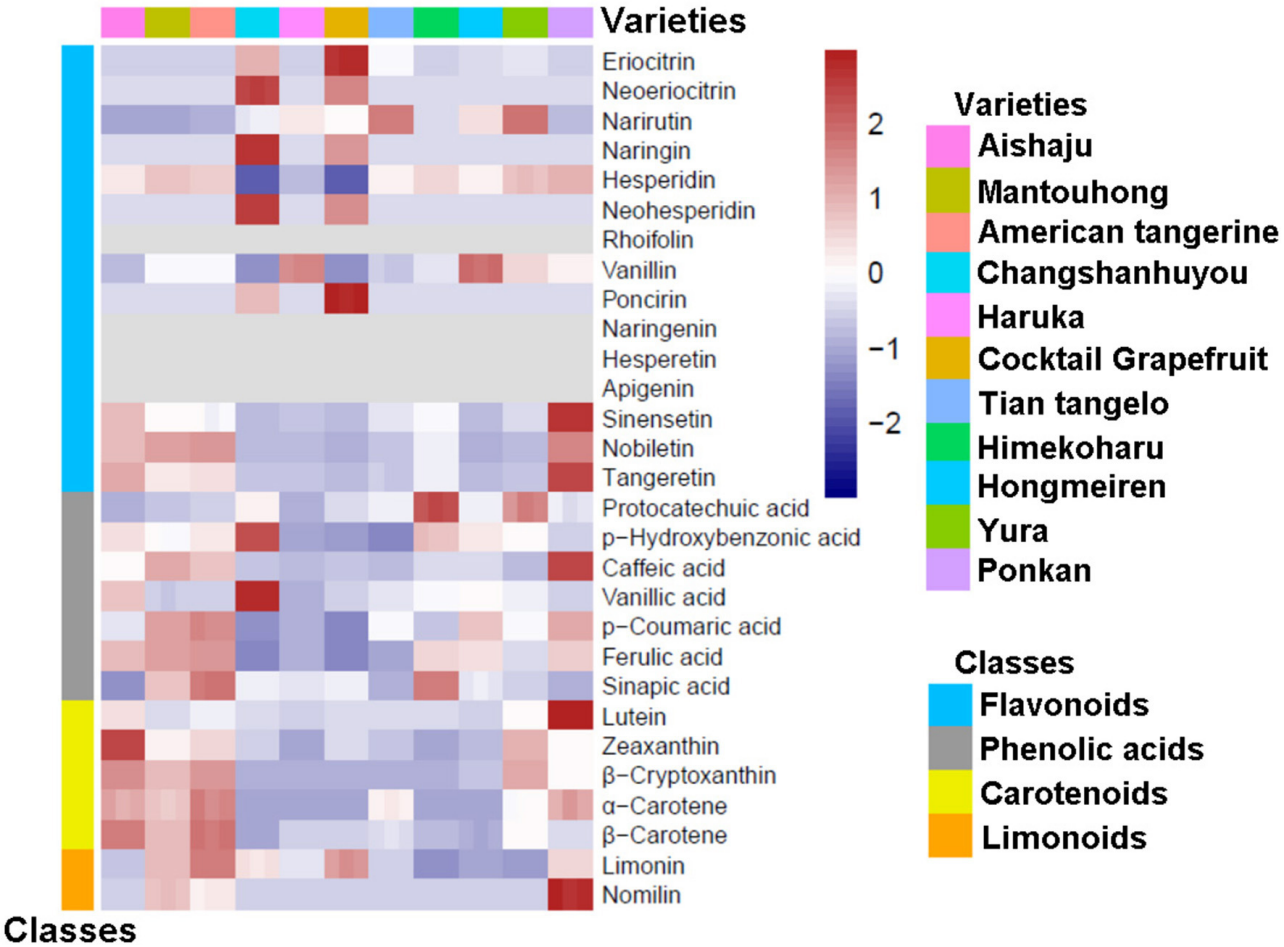
A heatmap of secondary metabolites in peel from different citrus varieties.

To study the components in the pulp and peel of various citrus varieties, we constructed heatmaps based on the relative variation of secondary metabolite contents among the 11 citrus cultivars (as shown in Figures 1, 2), and the contents of flavonoids, phenolic acids, carotenoids, and limonoids are summarized in Tables 1, 2. Moreover, each metabolite was quantified by the calibration curve of its corresponding standard in Supplementary Table 2.

**Table 1:** The contents of flavonoids, phenolic acids, carotenoids, and limonoids in citrus pulp (mg/kg FW).

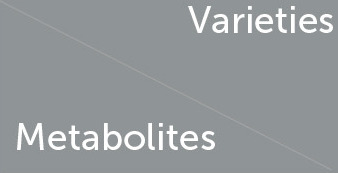	**Aishaju**	**Mantouhong**	**American tangerine**	**Changshanhuyou**	**Haruka**	**Cocktail Grapefruit**	**Tian tangelo**	**Himekoharu**	**Hongmeiren**	**Yura**	**Ponkan**
Eriocitrin (1)	0.44 ± 0.01f	0.52 ± 0.01f	1.02 ± 0.02ef	39.62 ± 0.40b	0.91 ± 0.01ef	46.42 ± 1.20a	1.50 ± 0.04de	2.00 ± 0.00cd	0.44 ± 0.01f	2.34 ± 0.07c	1.02 ± 0.02ef
Neoeriocitrin (2)	0.41 ± 0.02de	0.54 ± 0.07cde	0.41 ± 0.055de	31.17 ± 0.64b	nd	40.42 ± 0.01a	0.50 ± 0.04cde	0.48 ± 0.02cde	0.72 ± 0.08cd	0.78 ± 0.01c	0.40 ± 0.01de
Narirutin (3)	28.93 ± 0.18k	53.54 ± 0.21j	77.75 ± 0.01g	143.45 ± 0.55d	102.15 ± 0.42f	107.70 ± 0.21e	168.66 ± 0.45c	205.43 ± 0.24b	70.15 ± 0.42h	249.90 ± 1.30a	55.89 ± 0.04i
Naringin (4)	nd	nd	nd	313.11 ± 0.06a	nd	243.45 ± 0.48b	nd	nd	nd	nd	nd
Hesperidin (5)	522.30 ± 3.88e	779.41 ± 0.68c	816.33 ± 2.25b	10.70 ± 0.43j	120.98 ± 0.12i	6.58 ± 0.04k	185.83 ± 0.41g	865.50 ± 0.22a	161.34 ± 0.03h	575.96 ± 0.17d	490.36 ± 0.25f
Neohesperidin (6)	nd	nd	nd	145.34 ± 0.41b	nd	185.38 ± 0.13a	nd	nd	nd	nd	nd
Vanillin (7)	31.75 ± 0.31g	54.69 ± 0.24e	72.53 ± 0.09b	nd	62.23 ± 0.06d	nd	19.50 ± 0.20i	75.90 ± 0.67a	50.46 ± 0.28f	67.27 ± 0.02c	30.88 ± 0.10h
Poncirin (8)	nd	nd	nd	2.74 ± 0.13b	nd	8.29 ± 0.36a	nd	nd	nd	nd	nd
Naringenin (9)	nd	nd	nd	nd	nd	nd	nd	nd	nd	nd	nd
Hesperetin (10)	0.24 ± 0.01b	nd	nd	nd	nd	nd	nd	nd	nd	nd	0.43 ± 0.03a
**ΣFlavanones**	**584.07** **±3.98g**	**888.71** **±1.05d**	**968.04** **±2.29b**	**686.12** **±1.84e**	**286.27** **±0.60j**	**638.24** **±1.45f**	**375.98** **±1.06i**	**1,149.30** **±1.12a**	**283.11** **±0.82j**	**896.24** **±1.39c**	**578.97** **±0.39h**
Rhoifolin (11)	nd	nd	nd	nd	nd	nd	nd	nd	nd	nd	nd
Apigenin (12)	nd	nd	nd	nd	nd	nd	nd	nd	nd	nd	nd
**ΣFlavones**	**nd**	**nd**	**nd**	**nd**	**nd**	**nd**	**nd**	**nd**	**nd**	**nd**	**nd**
Sinensetin (13)	nd	nd	nd	nd	nd	nd	nd	nd	nd	nd	nd
Nobiletin (14)	0.29 ± 0.01c	0.18 ± 0.01e	0.68 ± 0.04b	0.07 ± 0.00f	0.07 ± 0.01f	0.26 ± 0.00d	0.27 ± 0.00cd	0.20 ± 0.00e	nd	nd	0.91 ± 0.00a
Tangeretin (15)	0.13 ± 0.01c	0.08 ± 0.00e	0.37 ± 0.00b	0.07 ± 0.00f	nd	0.13 ± 0.00c	0.10 ± 0.00d	0.08 ± 0.00e	nd	nd	0.50 ± 0.02a
**ΣPMFs**	**0.42** **±0.02c**	**0.25** **±0.01d**	**1.05** **±0.04b**	**0.14** **±0.01e**	**0.07** **±0.01f**	**0.40** **±0.00c**	**0.37** **±0.00c**	**0.27** **±0.00d**	**nd**	**nd**	**1.41** **±0.02a**
**ΣFlavonoids**	**584.49** **±3.96g**	**888.97** **±1.06d**	**969.08** **±2.32b**	**686.26** **±1.84e**	**286.33** **±0.59j**	**638.64** **±1.45f**	**376.35** **±1.06i**	**1,149.58** **±1.12a**	**283.11** **±0.82k**	**896.24** **±1.39c**	**580.39** **±0.41h**
Protocatechuic acid (16)	0.14 ± 0.00g	0.12 ± 0.00h	0.20 ± 0.00c	0.21 ± 0.00c	0.11 ± 0.01i	0.22 ± 0.00b	0.10 ± 0.01j	0.17 ± 0.00f	0.18 ± 0.00e	0.36 ± 0.00a	0.18 ± 0.00d
*p*-hydroxybenzonic acid (17)	1.55 ± 0.01c	1.05 ± 0.01e	0.99 ± 0.01g	1.38 ± 0.01d	0.69 ± 0.00i	0.57 ± 0.01j	0.38 ± 0.00k	2.09 ± 0.00b	1.02 ± 0.00f	2.16 ± 0.01a	0.82 ± 0.00h
Caffeic acid (18)	16.13 ± 0.04c	25.98 ± 0.06b	27.12 ± 0.17a	1.95 ± 0.01j	2.39 ± 0.01i	6.33 ± 0.03f	0.63 ± 0.00k	5.11 ± 0.06h	5.85 ± 0.02g	6.89 ± 0.00e	9.09 ± 0.01d
Vanillic acid (19)	2.89 ± 0.01d	1.80 ± 0.01h	1.67 ± 0.00i	3.53 ± 0.02b	1.52 ± 0.00k	4.11 ± 0.01a	2.84 ± 0.00e	2.68 ± 0.02f	1.64 ± 0.01j	3.49 ± 0.00c	2.33 ± 0.01g
*p*-coumaric acid (20)	3.88 ± 0.01f	5.40 ± 0.01e	5.37 ± 0.03e	2.15 ± 0.00h	6.90 ± 0.01b	1.42 ± 0.00j	1.75 ± 0.00i	5.74 ± 0.00d	8.06 ± 0.00a	6.79 ± 0.02c	2.94 ± 0.05g
Ferulic acid (21)	115.15 ± 0.02a	115.38 ± 0.04a	112.35 ± 0.04a	14.86 ± 0.03h	39.77 ± 0.03f	24.25 ± 0.00g	41.00 ± 0.07f	70.18 ± 0.03c	57.26 ± 0.01e	74.13 ± 0.02b	69.04 ± 0.15d
Sinapic acid (22)	6.06 ± 0.02e	8.03 ± 0.03d	9.27 ± 0.00c	5.64 ± 0.02g	1.55 ± 0.02k	10.37 ± 0.00b	2.45 ± 0.01j	11.92 ± 0.01a	5.71 ± 0.05f	3.95 ± 0.01i	4.99 ± 0.05h
**ΣPhenolic acids**	**145.79** **±0.08b**	**157.76** **±0.08a**	**156.97** **±0.24a**	**29.72** **±0.11h**	**52.93** **±0.06f**	**47.28** **±0.03g**	**49.16** **±0.06g**	**97.88** **±0.05c**	**79.72** **±0.04e**	**97.77** **±0.00c**	**89.39** **±0.27d**
Lutein (23)	0.54 ± 0.02a	0.21 ± 0.01e	0.31 ± 0.00c	0.09 ± 0.01h	0.04 ± 0.00j	0.11 ± 0.00g	0.13 ± 0.00f	nd	0.05 ± 0.00i	0.39 ± 0.01b	0.23 ± 0.00d
Zeaxanthin (24)	2.52 ± 0.00a	1.59 ± 0.02b	1.11 ± 0.01e	0.45 ± 0.01f	nd	0.38 ± 0.01g	0.44 ± 0.01f	0.05 ± 0.00i	0.23 ± 0.01h	1.36 ± 0.01c	1.17 ± 0.02d
β-cryptoxanthin (25)	6.48 ± 0.01c	9.02 ± 0.02b	4.98 ± 0.02d	0.10 ± 0.00h	nd	0.10 ± 0.01h	0.22 ± 0.01g	0.23 ± 0.00g	1.25 ± 0.01f	11.85 ± 0.00a	4.09 ± 0.03e
α-carotene (26)	0.07 ± 0.00a	nd	nd	nd	nd	nd	nd	nd	nd	0.06 ± 0.00b	nd
β-carotene (27)	0.30 ± 0.00a	0.25 ± 0.01c	0.16 ± 0.01d	nd	nd	nd	nd	nd	nd	0.27 ± 0.01b	0.14 ± 0.00e
**ΣCarotenoids**	**9.91** **±0.02c**	**11.08** **±0.01b**	**6.56** **±0.00d**	**0.63** **±0.02h**	**0.04** **±0.00k**	**0.59** **±0.01i**	**0.79** **±0.02g**	**0.28** **±0.00j**	**1.54** **±0.01f**	**13.92** **±0.03a**	**5.64** **±0.05e**
Limonin (28)	3.22 ± 0.31h	25.42 ± 0.99b	27.04 ± 0.10a	25.46 ± 0.03b	16.67 ± 0.04c	13.81 ± 0.15e	8.39 ± 0.03f	5.22 ± 0.32g	15.48 ± 0.80d	nd	17.45 ± 0.47c
Nomilin (29)	nd	5.35 ± 0.34c	4.49 ± 0.41d	nd	5.98 ± 0.14b	nd	nd	nd	4.51 ± 0.39d	nd	13.09 ± 0.87a
**ΣLimonoids**	**3.22** **±0.31h**	**30.77** **±0.65a**	**31.54** **±0.51a**	**25.46** **±0.02b**	**22.66** **±0.18c**	**13.81** **±0.15e**	**8.39** **±0.03f**	**5.22** **±0.32g**	**19.99** **±1.19d**	**nd**	**30.53** **±1.34a**

The content was represented as mean ± standard deviation (n = 3), and the significant differences were indicated by different letters in the same row (p < 0.05). Undetected was indicated by “nd.” Bold text indicates the name of a broad category of standards.

**Table 2:** The contents of flavonoids, phenolic acids, carotenoids, and limonoids in citrus peel (mg/kg FW).

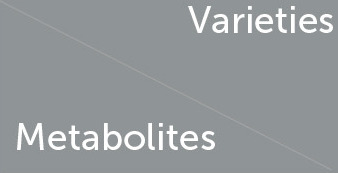	**Aishaju**	**Mantouhong**	**American tangerine**	**Changshanhuyou**	**Haruka**	**Cocktail Grapefruit**	**Tian tangelo**	**Himekoharu**	**Hongmeiren**	**Yura**	**Ponkan**
Eriocitrin (1)	0.69 ± 0.04i	3.24 ± 0.14gh	4.47 ± 0.06fg	130.48 ± 0.52b	4.58 ± 0.14fg	276.99 ± 3.84a	40.83 ± 0.83c	2.03 ± 0.09hi	7.00 ± 0.06e	17.92 ± 0.88d	4.70 ± 0.06fg
Neoeriocitrin (2)	2.77 ± 0.27ef	13.09 ± 1.41c	13.66 ± 0.33c	629.90 ± 7.14a	2.87 ± 0.09ef	436.33 ± 4.45b	3.14 ± 0.07ef	1.72 ± 0.05f	1.05 ± 0.04f	1.13 ± 0.02f	6.48 ± 0.03de
Narirutin (3)	25.08 ± 2.25k	43.52 ± 1.83j	61.75 ± 1.69i	287.23 ± 10.21f	468.56 ± 4.27d	366.19 ± 0.84e	925.49 ± 6.83b	210.33 ± 0.11g	505.93 ± 5.31c	953.80 ± 3.84a	125.90 ± 5.77h
Naringin (4)	nd	nd	nd	4,257.82 ± 4.90a	nd	2,484.86 ± 8.23b	nd	nd	nd	nd	nd
Hesperidin (5)	4,298.16 ± 2.26f	5,077.61 ± 14.42c	4,839.33 ± 38.30d	187.52 ± 1.59j	2,153.42 ± 13.92i	52.13 ± 2.24k	4,034.33 ± 1.96g	4,648.99 ± 7.36e	3,913.96 ± 4.99h	5,241.58 ± 96.21b	5,685.20 ± 0.22a
Neohesperidin (6)	nd	nd	nd	3,666.74 ± 16.17a	nd	2,280.04 ± 4.13b	nd	nd	nd	nd	nd
Vanillin (7)	67.31 ± 0.59h	166.77 ± 1.66e	166.61 ± 2.47e	nd	394.59 ± 4.85b	nd	89.41 ± 0.91g	128.05 ± 0.58f	443.41 ± 7.76a	245.63 ± 2.61c	206.00 ± 0.26d
Poncirin (8)	nd	nd	nd	36.53 ± 0.60b	nd	90.91 ± 1.13a	nd	nd	nd	nd	nd
Naringenin (9)	nd	nd	nd	nd	nd	nd	nd	nd	nd	nd	nd
Hesperetin (10)	nd	nd	nd	nd	nd	nd	nd	nd	nd	nd	nd
**ΣFlavanones**	**4,394.01** **±0.83h**	**5,304.22** **±16.35d**	**5,085.83** **±34.41e**	**9,196.22** **±41.13a**	**3,024.02** **±23.27i**	**5,987.44** **±8.18c**	**5,093.21** **±8.78e**	**4,991.11** **±7.90f**	**4,871.34** **±18.08g**	**6,460.06** **±103.56b**	**6,028.28** **±5.85c**
Rhoifolin (11)	nd	nd	nd	nd	nd	nd	nd	nd	nd	nd	nd
Apigenin (12)	nd	nd	nd	nd	nd	nd	nd	nd	nd	nd	nd
**ΣFlavones**	**nd**	**nd**	**nd**	**nd**	**nd**	**nd**	**nd**	**nd**	**nd**	**nd**	**nd**
Sinensetin (13)	39.13 ± 0.04b	19.21 ± 0.80c	16.03 ± 1.42e	0.66 ± 0.06i	3.24 ± 0.14h	nd	9.88 ± 0.271f	17.29 ± 0.06d	nd	6.90 ± 0.06g	79.24 ± 0.24a
Nobiletin (14)	688.48 ± 0.06d	798.82 ± 0.55c	836.81 ± 0.73b	44.99 ± 0.18h	42.98 ± 0.11i	23.70 ± 0.12j	90.39 ± 1.10f	301.10 ± 0.74e	4.35 ± 0.28k	55.72 ± 0.20g	928.54 ± 0.93a
Tangeretin (15)	184.70 ± 0.27b	106.25 ± 0.55d	111.96 ± 0.06c	13.65 ± 0.06g	12.60 ± 0.04gh	6.90 ± 0.04j	19.74 ± 0.49f	62.58 ± 0.02e	1.27 ± 0.06k	9.35 ± 0.10i	310.47 ± 0.10a
**ΣPMFs**	**912.30** **±0.37d**	**924.27** **±1.91c**	**964.79** **±2.09b**	**59.30** **±0.18h**	**58.82** **±0.29h**	**30.60** **±0.09i**	**120.02** **±0.87f**	**380.97** **±0.83e**	**5.62** **±0.22j**	**71.96** **±0.24g**	**1,318.25** **±0.59a**
**ΣFlavonoids**	**5,306.31** **±1.20g**	**6,228.50** **±14.44d**	**6,050.62** **±32.33e**	**9,255.52** **±41.31a**	**3,082.83** **±22.97j**	**6,018.05** **±8.09e**	**5,213.23** **±9.65h**	**5,372.08** **±7.07f**	**4,876.96** **±17.85i**	**6,532.02** **±103.32c**	**7,346.53** **±5.26b**
Protocatechuic acid (16)	1.01 ± 0.01h	1.23 ± 0.03g	1.31 ± 0.01g	1.90 ± 0.00c	1.02 ± 0.01h	1.40 ± 0.00ef	1.61 ± 0.03d	3.63 ± 0.09a	1.62 ± 0.00d	3.06 ± 0.150b	1.48 ± 0.10e
*p*-hydroxybenzonic acid (17)	13.52 ± 0.02c	11.12 ± 0.04f	12.59 ± 0.01d	24.12 ± 0.01a	5.35 ± 0.07h	4.87 ± 0.00i	3.33 ± 0.04j	15.89 ± 0.03b	12.58 ± 0.03d	11.43 ± 0.06e	7.75 ± 0.01g
Caffeic acid (18)	61.38 ± 0.24d	141.56 ± 0.28b	113.05 ± 0.41c	10.22 ± 0.16g	1.02 ± 0.01j	9.05 ± 0.04h	0.73 ± 0.00j	29.54 ± 0.11e	26.45 ± 0.28f	4.81 ± 0.00i	237.46 ± 0.15a
Vanillic acid (19)	24.30 ± 0.08b	14.13 ± 0.06i	14.81 ± 0.10g	40.32 ± 0.21a	11.75 ± 0.14j	14.17 ± 0.08i	16.31 ± 0.00f	18.07 ± 0.03d	19.52 ± 0.00c	17.34 ± 0.02e	14.49 ± 0.03h
*p*-coumaric acid (20)	56.02 ± 0.03f	125.98 ± 0.15b	137.97 ± 0.51a	17.41 ± 0.00i	32.18 ± 0.00h	11.62 ± 0.00j	67.21 ± 0.07e	43.25 ± 0.09g	105.32 ± 0.82d	67.08 ± 0.02e	118.79 ± 0.03c
Ferulic acid (21)	778.91 ± 0.14c	904.96 ± 0.02b	919.51 ± 0.08a	124.64 ± 0.37j	255.64 ± 0.27h	109.89 ± 0.25k	209.87 ± 0.07i	695.18 ± 0.53e	645.44 ± 0.34f	408.08 ± 0.14g	706.23 ± 2.82d
Sinapic acid (22)	1.44 ± 0.04h	64.33 ± 0.28c	95.77 ± 1.91a	32.09 ± 0.37d	30.77 ± 0.13e	31.93 ± 0.13d	12.53 ± 0.03g	93.55 ± 0.01b	31.23 ± 0.39de	22.18 ± 0.00f	12.29 ± 0.55g
**ΣPhenolic acids**	**936.59** **±0.35d**	**1,263.31** **±0.25b**	**1,295.02** **±0.80a**	**250.71** **±0.04j**	**337.72** **±0.10h**	**182.94** **±0.01k**	**311.57** **±0.12i**	**899.10** **±0.36e**	**842.17** **±1.03f**	**534.00** **±0.02g**	**1,098.50** **±3.60c**
Lutein (23)	2.03 ± 0.01b	0.60 ± 0.00e	0.98 ± 0.01d	0.40 ± 0.00g	0.27 ± 0.00h	0.40 ± 0.00g	0.41 ± 0.00g	0.52 ± 0.01f	0.26 ± 0.01h	1.39 ± 0.00c	6.86 ± 0.00a
Zeaxanthin (24)	12.63 ± 0.01a	4.32 ± 0.01d	5.74 ± 0.03c	1.96 ± 0.01g	0.12 ± 0.00k	2.38 ± 0.00f	1.51 ± 0.01h	0.26 ± 0.01j	0.99 ± 0.00i	7.58 ± 0.02b	3.85 ± 0.00e
β-cryptoxanthin (25)	26.17 ± 0.05a	20.06 ± 0.01d	25.12 ± 0.05b	0.36 ± 0.01i	0.14 ± 0.01k	0.24 ± 0.00j	0.51 ± 0.01g	0.45 ± 0.00h	3.24 ± 0.01f	22.07 ± 0.01c	10.42 ± 0.00e
α-carotene (26)	0.11 ± 0.00b	0.09 ± 0.002c	0.13 ± 0.01a	nd	nd	nd	0.06 ± 0.00d	nd	nd	0.05 ± 0.00d	0.11 ± 0.01b
β-carotene (27)	0.63 ± 0.00b	0.47 ± 0.00c	0.65 ± 0.01a	0.07 ± 0.00k	0.18 ± 0.00g	0.16 ± 0.00h	0.20 ± 0.00e	0.13 ± 0.00i	0.09 ± 0.01j	0.30 ± 0.00d	0.19 ± 0.00f
**ΣCarotenoids**	**41.57** **±0.05a**	**25.54** **±0.03d**	**32.61** **±0.04b**	**2.79** **±0.00h**	**0.70** **±0.01k**	**3.19** **±0.01g**	**2.69** **±0.00i**	**1.35** **±0.00j**	**4.58** **±0.00f**	**31.39** **±0.02c**	**21.43** **±0.01e**
Limonin (28)	110.68 ± 0.33h	269.18 ± 2.84c	356.63 ± 1.94a	214.14 ± 1.58e	144.78 ± 1.34f	324.64 ± 15.59b	119.70 ± 1.61g	37.38 ± 1.79k	71.51 ± 0.61i	49.07 ± 0.59j	227.79 ± 1.018d
Nomilin (29)	nd	47.94 ± 1.29b	28.00 ± 1.25c	nd	nd	nd	nd	nd	nd	nd	122.83 ± 4.91a
**ΣLimonoids**	**110.68** **±0.33g**	**317.12** **±4.13c**	**384.63** **±3.19a**	**214.14** **±1.58d**	**144.78** **±1.34e**	**324.64** **±15.59c**	**119.70** **±1.61f**	**37.38** **±1.79j**	**71.51** **±0.61h**	**49.07** **±0.59i**	**350.62** **±3.90b**

The content was represented as mean ± standard deviation (n = 3), and the significant differences were indicated by different letters in the same row (p < 0.05). Undetected was indicated by “nd.” Bold text indicates the name of a broad category of standards.

### 3.1. Identification of components in citrus pulp

Pulp was the main consumable part of citrus, which directly provided nutritional and bioactive components to the human body. In our research, the flavonoid contents in citrus pulp ranged from 283.11 to 1,149.58 mg/kg fresh weight (FW), which were dominated by flavanones with a minimal content of PMFs, and no flavones were detected (as seen in Table 1 and Figure 1). In addition, no naringenin (9) or sinensetin (13) were found in the pulp of all 11 citrus varieties. There were significant differences among varieties; the lowest flavonoid content in pulp was found in Hongmeiren, while Himekoharu contained the highest content, which was approximately 4 times that of the former. Except for Changshanhuyou and cocktail grapefruit, hesperidin (5) was the main ingredient that dominated more than 42% of the flavonoid contents. In terms of its content, narirutin (3) was another major component, and it varied significantly among varieties (as Figure 1 displays), with the highest and second-highest being Yura (249.90 mg/kg FW) and Himekoharu (205.43 mg/kg FW), and the lowest and second-lowest being Aishaju (28.93 mg/kg FW) and Mantouhong (53.54 mg/kg FW), of which the highest was approximately 8.6-fold higher than the lowest. Vanillin (7) was also one of the major flavonoids among varieties, except that Changshanhuyou and cocktail grapefruit were undetected, and their contents were between 50.46 mg/kg FW and 75.90 mg/kg FW. In contrast, naringin (4), neohesperidin (6), and poncirin (8) were exclusive to Changshanhuyou and cocktail grapefruit; none of the three flavonoids had been found in other varieties. In addition, naringin (4) and neohesperidin (6) were the most abundant flavonoids in Changshanhuyou (311.11 mg/kg FW and 145.34 mg/kg FW, respectively) and cocktail grapefruit (243.45 mg/kg FW and 185.38 mg/kg FW, respectively), while poncirin (8) was the least abundant with the contents of only 2.74 mg/kg FW and 8.29 mg/kg FW, respectively. Besides, among the varieties, cocktail grapefruit showed the highest content of eriocitrin [(1), 46.42 mg/kg FW] and neoeriocitrin [(2), 40.42 mg/kg FW], followed by Changshanhuyou with contents of 39.62 mg/kg FW and 31.17 mg/kg FW, respectively. However, these two components were less abundant in the other nine varieties, where the eriocitrin (1) contents were only 0.44 to 2.34 mg/kg FW and all contents of neoeriocitrin (2) were <1 mg/kg FW. The least content of hesperetin (10) was only found in Aishaju and Ponkan, with contents of 0.24 mg/kg FW and 0.43 mg/kg FW, respectively. As for nobiletin (14) and tangeretin (15), although these two PMFs were detectable in most varieties, they were both present at diminutive levels, and the highest contents of nobiletin [(14), 0.91 mg/kg FW] and tangeretin [(15), 0.50 mg/kg FW] were found in Ponkan.

A total of seven phenolic acids were quantified in the pulp extracts, and the content of total phenolic acids ranged from 29.72 to 157.76 mg/kg FW. Overall, ferulic acid (21) was the main phenolic acid in all varieties, which occupied over 50% of the total phenolic acid content for each species, and the highest contents were identified in Mantouhong (115.38 mg/kg FW), Aishaju (115.15 mg/kg FW), and American tangerine (112.35 mg/kg FW), while the lowest content was identified in Changshanhuyou (only 14.86 mg/kg FW). In contrast, small amounts of protocatechuic acid (16) were present in all cultivars, ranging from 0.10 to 0.36 mg/kg FW. Moreover, low levels of phydroxybenzonic acid [(17), 0.38–2.16 mg/kg FW], vanillic acid [(19), 1.52–4.11 mg/kg FW], *p*-coumaric acid [(20), 1.42–8.06 mg/kg FW], and sinapic acid [(22), 1.55–11.92 mg/kg FW] were contained in the pulp. Besides, it should be noted that there was a significant difference among varieties: moderate levels of caffeic acid (18) were detected in American tangerine (27.12 mg/kg FW), Mantouhong (25.98 mg/kg FW), and Aishaju (16.13 mg/kg FW), while low levels were detected in the remaining varieties. In the pulp, the phenolic acids were rich in American tangerine, Mantouhong, and Aishaju but poor in Changshanhuyou (seen in Table 1).

As a whole, the carotenoid content in citrus pulp was found to be low (0.04–13.92 mg/kg FW). In detail, the quantities of lutein (23), α-carotene (26), and β-carotene (27) were negligible, and the highest contents of lutein (23) and β-carotene (27) were found in Aishaju, at 0.54 mg/kg FW and 0.30 mg/kg FW, respectively, whereas α-carotene (26) was undetectable in most varieties. Zeaxanthin (24) was more abundant in Aishaju than the above three, and the highest content was 2.52 mg/kg FW in Aishaju. Among these carotenoids, β-cryptoxanthin (25) was considered to be the major component, but its content varied from 0.1 to 11.85 mg/kg FW. The highest content of β-cryptoxanthin (25) was found in Yura, followed by Mantouhong, Aishaju, American tangerine, and Ponkan, except Haruka, which was undetected; all these species had orange-yellow pulp, while a marginal amount of β-cryptoxanthin (25) resulted in the pulp of Changshanhuyou, cocktail grapefruit, Tian tangelo, and Himekoharu turning yellow-green. Furthermore, as indicated by the heatmap in Figure 1, Yura and Mantouhong were different from others and were richer in carotenoids when combined with the data in Table 1.

Limonoids, including limonin (28) and nomilin (29), were measured in the citrus pulp. By comparison, no limonoids were detected in Yura, whereas the limonoid content in the remaining varieties ranged between 3.22 mg/kg FW and 27.94 mg/kg FW, and it is worth mentioning that all varieties had higher limonin (28) content than nomilin (29). For limonin (28), the highest and lowest contents corresponded to American tangerine and Aishaju, followed by Mantouhong (25.42 mg/kg FW) and Changshanhuyou (25.46 mg/kg FW), both of which had no obvious difference. As for nomilin (29), there was a notable difference among varieties: nomilin was the most abundant limonoid in Ponkan (29) with a content of 13.09 mg/kg FW, and the content of nomilin in Mantouhong, American tangerine, Haruka, and Hongmeiren was ~5 mg/kg FW, while the rest of the varieties remained unidentified.

### 3.2. Determination of compositions and contents of citrus peels

As the inedible part, the peel is the major by-product of the citrus industry, and only a small portion of the peel is used for commercial processing; most of the peels are discarded and become contaminants. However, citrus peels contain valuable phytochemicals and are sources of bioactive ingredients. Thus, the composition and contents of citrus peels were thoroughly analyzed in this study (as shown in Figure 2 and Table 2). Citrus peels contain abundant flavonoids, with contents ranging from 3,082.83 to 9,255.52 mg/kg FW. The main flavonoids in the peel are flavanones, followed by PMFs, with no flavones found. As the main flavanone, hesperidin (5) ranged from 2,153.42 to 5,685.20 mg/kg FW, except in Changshanhuyou and cocktail grapefruit. In addition, the highest contents of narirutin (3) and vanillin (7) were identified in Yura (953.80 mg/kg FW) and Hongmeiren (443.41 mg/kg FW), respectively. However, naringenin (9) and hesperetin (10) were undetectable in all varieties. For the remaining five flavanones, the contents of Changshanhuyou and cocktail grapefruit showed significant differences compared to the other nine species, as shown in Figure 2 and Table 2. Similar to the pulp, naringin (4), neohesperidin (6), and poncirin (8) were only found in Changshanhuyou and cocktail grapefruit. Furthermore, naringin (4), and neohesperidin (6) were the two most abundant metabolites, with the highest levels of 4,257.82 and 3,666.74 mg/kg FW in Changshanhuyou and of 2,484.86 and 2,280.04 mg/kg FW in cocktail grapefruit, respectively. In contrast, the contents of poncirin (8) were much lower only at 36.53 and 90.91 mg/kg FW in Changshanhuyou and cocktail grapefruit. In addition, cocktail grapefruit contained the most content of eriocitrin [(1), 276.99 mg/kg FW], and Changshanhuyou had the highest neoeriocitrin content [(2), 629.90 mg/kg FW]. In terms of PMFs, the contents of the peel were much higher than that of the pulp, and they were enriched in Ponkan (1,318.25 mg/kg FW), American tangerine (964.79 mg/kg FW), Mantouhong (924.27 mg/kg FW), and Aishaju (912.30 mg/kg FW). Nobiletin (14) was the main PMF detected in the peel samples. The contents of PMFs in Ponkan were higher than that in other varieties, and the contents of each component were 928.54 mg/kg FW [nobiletin (14)], 310.47 mg/kg FW [tangeretin (15)], and 79.24 mg/kg FW [sinensentin (13)].

The contents of phenolic acids in the peel ranged from 182.94 to 1,295.02 mg/kg FW, much higher than that in the pulp. In a word, American tangerine, Mantouhong, and Ponkan were rich in various phenolic acids, whereas cocktail grapefruit and Changshanhuyou were less abundant. Ferulic acid (21) was found to be the most common phenolic acid in citrus peels, with American tangerine having the highest content at 919.51 mg/kg FW and cocktail grapefruit having the lowest at 109.89 mg/kg FW. Protocatechuic acid (16) was present at trivial levels (1.01–3.63 mg/kg FW), and low levels of phydroxybenzonic acid [(17), 3.33–24.12 mg/kg FW], and vanillic acid [(19), 11.75–40.32 mg/kg FW] were detected, where the contents in Changshanhuyou were much higher than the remaining varieties (combined with the heatmap in Figure 2 and Table 2). The caffeic acid (18) content in the peel also varied significantly among varieties, and the highest concentration of caffeic acid (18) was in Ponkan at 237.46 mg/kg FW, while in other varieties it ranged from 0.73 to 113.05 mg/kg FW. In addition, the most abundant *p*-coumaric acid (20) and sinapic acid (22) were detected in American tangerine (137.97 mg/kg FW) and Himekoharu (93.55 mg/kg FW), respectively.

In terms of carotenoids, the peel had the lowest content (0.7–41.57 mg/kg FW) among the four types of metabolites, which was the same as the pulp. Lutein (23), α-carotene (26), and β-carotene (27) contents were present at minimal levels; β-carotene (27) content ranged between 0.07 and 0.63 mg/kg FW; α-carotene (26) was detected in six varieties at ~0.10 mg/kg FW or less; lutein (23) content was below 2 mg/kg FW except for Ponkan (6.86 mg/kg FW), which showed a great difference with the others. As shown in Figure 2, the zeaxanthin (24) content of Aishaju was much higher than that of the other varieties, and a notable difference could be found from the data in Table 2. β-cryptoxanthin (25) was the main carotenoid in most varieties, but its content varied with species. The most abundant species were Aishaju (26.17 mg/kg FW), American tangerine (25.12 mg/kg FW), Yura (22.07 mg/kg FW), Mantouhong (20.06 mg/kg FW), and Ponkan (10.42 mg/kg FW), which had high β-cryptoxanthin (25) content and orange-red peels. In contrast, the remaining species except Hongmeiren contained diminutive quantities of β-cryptoxanthin [(25), 0.14–0.51 mg/kg FW] with yellow-green peels. Notably, Hongmeiren had a low β-cryptoxanthin (25) content of 3.24 mg/kg FW but had orange-red skin.

The limonoids in the peel were far more abundant than those in the pulp, and their contents ranged from 37.38 to 384.63 mg/kg FW. Limonin (28) was the major component for all varieties, and American tangerine had the highest content of 356.63 mg/kg FW, and Hongmeiren had the lowest content of 37.38 mg/kg FW. Nomilin (29) was only detected in Ponkan (122.83 mg/kg FW), Mantouhong (47.94 mg/kg FW), and American tangerine (28.00 mg/kg FW), whereas Ponkan was significantly higher than the other two varieties.

### 3.3. Comparison of secondary metabolites in the pulp and peel

**Figure 3:**
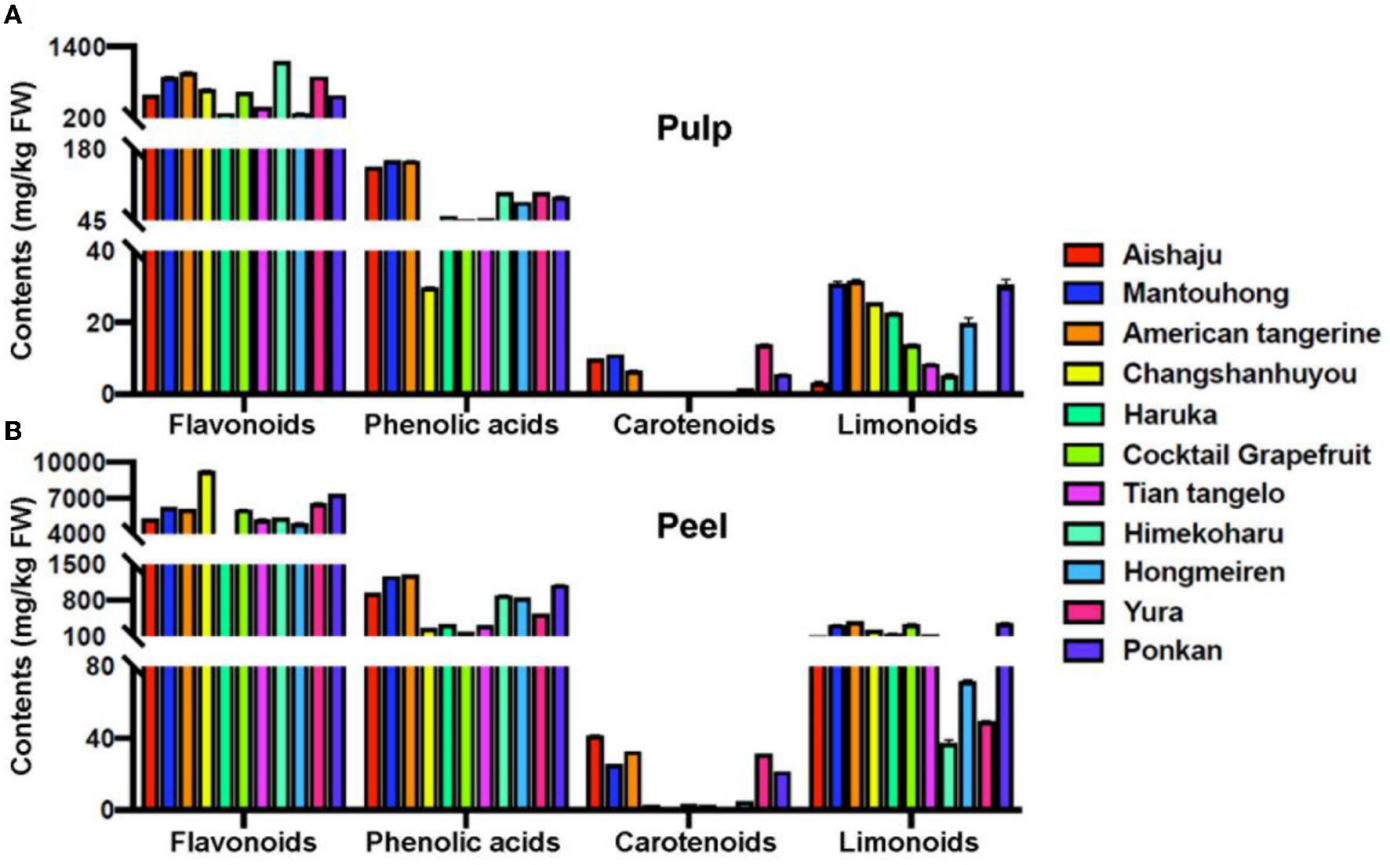
Total flavonoids, total phenolic acids, total carotenoids, and total limonoids of different citrus varieties in the pulp **(A)** and in the peel **(B)**.

To investigate the distribution of metabolites in different citrus varieties, the total accumulation of these secondary metabolites in the pulp and peel was further analyzed comparatively. As shown in Figure 3, the distribution pattern for these metabolites in most varieties is given below: flavonoids were the most abundant compounds, followed by phenolic acids, with carotenoids and limonoids being far less abundant than the first two, but limonoids were greater than carotenoids. However, there were still exceptions, such as the pulp of Aishaju and Yura, which contained more carotenoids than limonoids, and the peel of cocktail grapefruit, which had higher limonoid contents than phenolic acids. To indicate the difference in the total content of these metabolites in the pulp and peel, we defined the content ratio (CR) between peel and pulp as the difference in metabolite content, and the higher the CR value, the greater the difference in metabolite content, as shown in Table 3. The CR values of flavonoids in each citrus variety ranged from 4.67 to 13.85, with a mean value of 10.15 (Table 3), but the ranking of total accumulation in the peel and pulp was inconsistent among varieties, especially for the variety with high content. In the peel, the highest CR value was found in Changshanhuyou, followed by Ponkan and Yura, and the lowest was found in Haruka, which was much lower than the other varieties, whereas, in the pulp, the highest CR value was found in Himekoharu, followed by American tangerine and Mantouhong, and the lowest was found in Hongmeiren. The phenolic acid content also showed inconsistencies, with the CR values ranging from 3.87 to 12.29 and a mean value of 7.75. American tangerine and cocktail grapefruit had the highest and lowest contents in the peel, respectively, but Mantouhong, American tangerine, and Aishaju had the highest content and Changshanhuyou had the lowest content in the pulp. Carotenoids were the lowest in the peel and pulp of most varieties compared to other metabolites, and the CR values ranged from 2.26 to 17.50, and the mean value was 5.10. In the peel, Aishaju had the highest content of carotenoids, followed by American tangerine, Yura, Mantouhong, and Ponkan, and the rest of the varieties had the lowest content; however, the highest levels in the pulp were found in Yura, followed by Mantouhong and Aishaju, and most of the remaining varieties had marginal levels of carotenoids. In addition, the lowest levels of carotenoids were found in Haruka in both the peel and pulp. Similarly, the most abundant species of limonoids were American tangerine, Ponkan, and Mantouhong both in the peel and pulp. However, the accumulation was inconsistent at low levels; Himekoharu, Yura, and Hongmeiren had the lowest accumulation of limonoids in the peel but Yura and Aishaju in the pulp. The CR values for limonoids ranged from 3.58 to 34.37 (no value shown as Yura pulp was not detected), with the greatest variation and a mean value of 13.17. In a word, the total amount of these metabolites in the peel was much greater than in the pulp, and the accumulation varied significantly between species. Limonoids showed the greatest variation in CR values among varieties, while carotenoids were relatively low.

**Table 3:** The content ratio values of secondary metabolites in different citrus varieties.

**Varieties**	**Flavonoids**	**Phenolic acids**	**Carotenoids**	**Limonoids**
Aishaju	9.08	6.42	4.19	34.37
Mantouhong	7.01	8.01	2.31	10.31
American tangerine	6.24	8.25	4.97	12.19
Changshanhuyou	13.49	8.44	4.43	8.41
Haruka	10.77	6.38	17.5	6.39
Cocktail Grapefruit	9.42	3.87	5.41	23.51
Tian tangelo	13.85	6.34	3.41	14.27
Himekoharu	4.67	9.19	4.82	7.16
Hongmeiren	17.23	10.56	2.97	3.58
Yura	7.29	5.46	2.26	–
Ponkan	12.66	12.29	3.8	11.48
Mean	10.15	7.75	5.1	13.17

The content ratio values were metabolite content in the peel divided by metabolite content in the pulp.

### 3.4. Correlation between secondary metabolites in citrus

**Figure 4:**
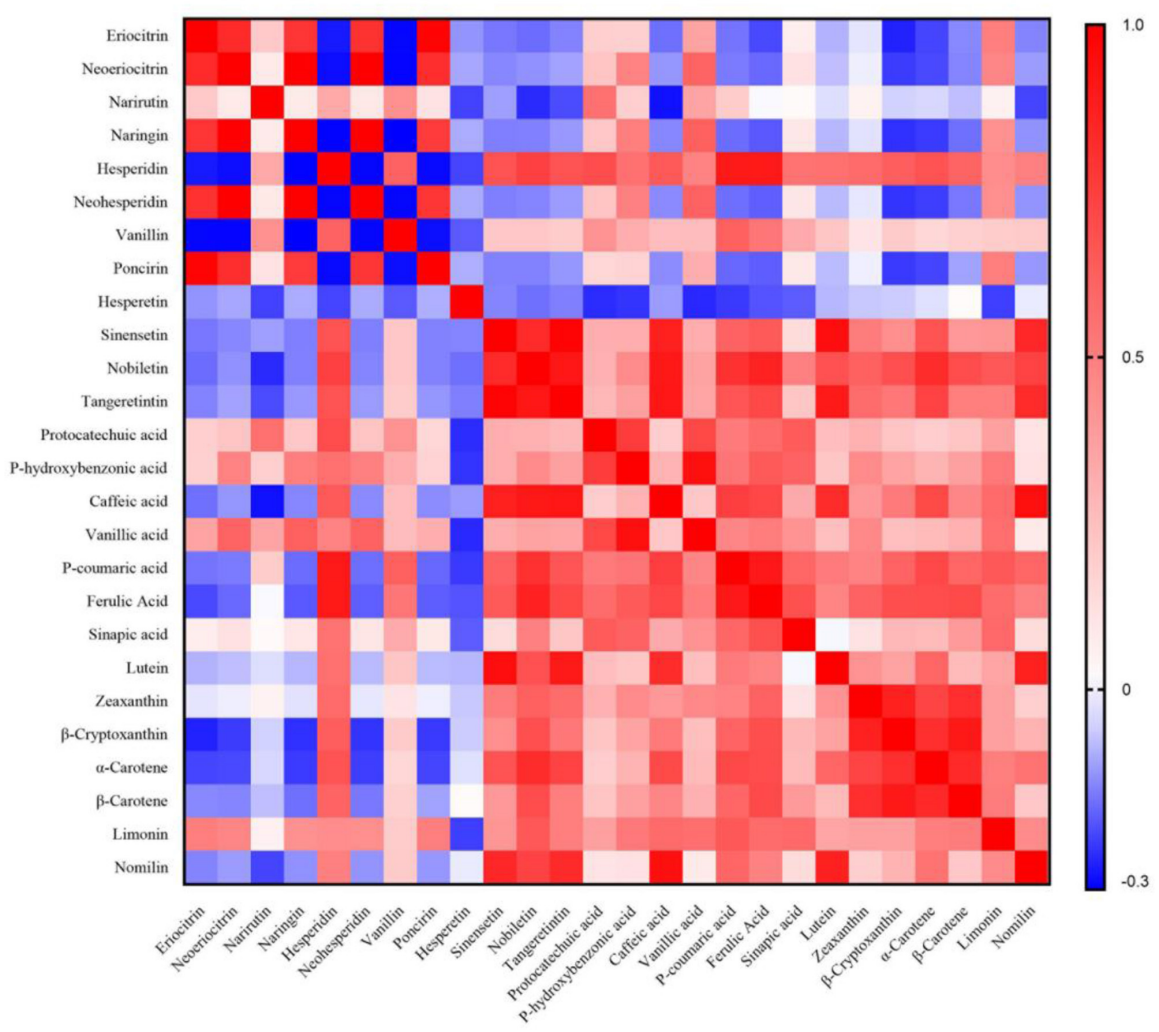
Correlation heatmap of secondary metabolites.

The relationships between secondary metabolite contents in 11 citrus varieties were thoroughly examined using the Pearson correlation analysis (Figure 4). The results indicated that positive correlations existed among PMFs, phenolic acids, carotenoids, and limonoids, but most of them were generally negatively correlated with flavanones. In particular, PMFs, caffeic acid (18), lutein (23), and nomilin (29) showed highly positive correlations with each other. As shown in Supplementary Table 3, there were significant relationships between zeaxanthin (24), β-cryptoxanthin (25), α-carotene (26), and β-carotene [(27), *r* ≥ 0.732, *p* < 0.05], and protocatechuic acid (16) was positively correlated with phydroxybenzonic acid [(17), *r* = 0.761, *p* < 0.05]. Similarly, for the flavanones, eriocitrin (1), neoeriocitrin (2), naringin (4), neohesperidin (6), and poncirin (8) had strong positive correlations with each other. However, hesperidin (5) showed negative associations with them but was positively associated with the remaining metabolites [not with hesperetin (10)]. Meanwhile, negative or no obvious correlations were observed between hesperetin (10) and other metabolites. These results revealed strong correlations between secondary metabolites in citrus and demonstrated that the above complex data could be further visualized using PCA.

### 3.5. PCA and HCA analysis

The citrus grouping and metabolite discrimination at the species and individual levels were important for breeding and resource utilization. Therefore, data on flavonoids, phenolic acids, carotenoids, and limonoids from 11 citrus varieties in the Zhejiang region were analyzed using PCA and HCA to explore their chemical composition-based classification and metabolite identification in citrus species, and analyses of these metabolites in citrus peel and pulp were separately performed.

#### 3.5.1. PCA and HCA analysis in the citrus pulp

**Figure 5:**
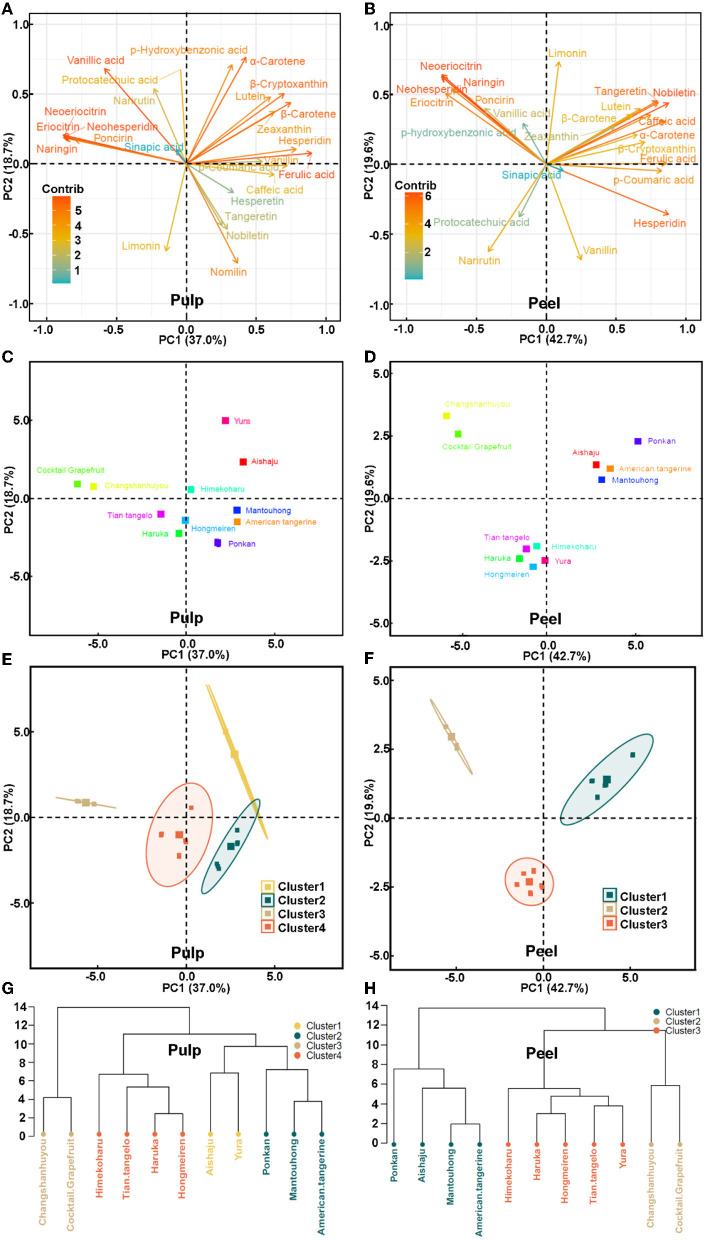
**(A, B)** Loading plots of principal components of the PCA results obtained from metabolite data in the pulp and peel. **(C, D)** Score plots of principal components from different citrus varieties in the pulp and peel. **(E, F)** The outliers of PCA in the pulp and peel. **(G, H)** Hierarchical clustering in the pulp and peel.

The cumulative percent variance (CPV) for the three principal variables in the pulp was calculated to be 73.4%, which meets the requirement of CPV over 70% for PCA analysis (23). The resulting data were drawn into a representative score plot of the two principal components (Figure 5A), where the first principal component (PC1) and the second principal component (PC2) accounted for 37.0 and 18.7% of the total variance, respectively. The contributors of PC1 and PC2 were compared. For PC1, the main metabolites with the most positive loadings were ferulic acid (21), hesperidin (5), β-carotene (27), vanillin (7), and β-cryptoxanthin (25), while those with the negative loadings were eriocitrin (1), neohesperidin (6), neoeriocitrin (2), naringin (4), and poncirin (8). For PC2, the most positive contributors were α-carotene (26), phydroxybenzonic acid (17), vanillic acid (19), and protocatechuic acid (16), while those with the most negative contributors turned out to be nomilin (29) and limonin (28). The distances between varieties in the score plots of the two principal components showed the composition differences of the main metabolites in the pulp for each citrus variety (Figure 5C). The similarity of metabolite compositions indicated the affinity between varieties, and the citrus varieties could be classified into four distinct groups (Figures 5E, G). Yura and Aishaju formed the first group, which were both in the first quadrant (Figure 5C), and all carotenoids also belonged in the first quadrant (Figure 5A); in addition, such a coincidence indicated that Yura and Aishaju had higher carotenoids in the pulp than other varieties. The next group was Changshanhuyou and cocktail grapefruit, and in Figure 5C, they were adjacent to each other, showing that these two varieties contained similar metabolite compositions. Coincidentally, eriocitrin (1), neohesperidin (6), neoeriocitrin (2), naringin (4), and poncirin (8) were also located in the same region (Figure 5A), and the results suggested that these metabolites were markers to discriminate Changshanhuyou and cocktail grapefruit from the other varieties. Mantouhong, American tangerine, and Ponkan were grouped, and the last group was formed by Haruka, Tian tangelo, Himekoharu, and Hongmeiren. Moreover, all varieties were ranked according to the scores of the four principal components (Supplementary Table 4), positive for the first five varieties, and negative for the last six varieties.

#### 3.5.2. PCA and HCA analyses in the citrus peel

The CPV for the three principal variables in the peel accounted for 73.3%, and the score plot for the two principal components is displayed in Figure 5B, where PC1 and PC2 explained 42.7 and 18.7% of data variability, respectively. As shown in Figure 5B, the metabolites with the most positive contribution for PC1 were nobiletin (14), hesperidin (6), ferulic acid (21), caffeic acid (18), and *p*-coumaric acid (20) and for PC2 were limonin (28), neoeriocitrin (2), and naringin (4), whereas neohesperidin (6), neoeriocitrin (2), naringin (4), eriocitrin (1), and poncirin (8) made the greatest negative contribution for PC1 and vanillin (7) and narirutin for PC2 (3). In contrast to the results in the pulp, these 11 citrus varieties were classified into three categories based on the similarity of metabolite compositions in the peel (Figures 5F, H). The first category was Aishaju, Mantouhong, American tangerine, and Ponkan. The same results were obtained as in the pulp, where Changshanhuyou and cocktail grapefruit belonged to the same category since they had similar metabolite compositions (Figure 5D). Haruka, Tian tangelo, Himekoharu, Hongmeiren, and Yura fell into the last category. In addition, as summarized in Supplementary Table 3, all varieties were ranked based on the scores of four principal components, with the first four varieties being positive and the last seven varieties being negative.

## 4. Discussion

This study presented comprehensive research on the distribution pattern of citrus secondary metabolites and their variability among different varieties. The study explored the chemical composition-based classification and metabolite identification of citrus varieties by measuring secondary metabolites in citrus pulp and peel and further analyzing the data by PCA and HCA.

Flavonoids are a large group of compounds synthesized by plants and share a basic chemical structure (24). Citrus fruits are abundant in flavonoids, and recent literature mainly focused on the study of flavonoids in the peel or different citrus varieties (11, 25); however, there were also differences observed in the compositions and contents of flavonoids between different tissue parts in the same citrus variety (21, 26). Wang et al. (13) reported similar results to ours, namely that citrus peels were far more abundant in flavonoids than the pulp. Flavanones constituted the majority of flavonoids in citrus fruits; Peterson et al. (27) reported similar results that flavanones were the main flavonoids in mandarins, tangors, and tangelos. In addition, the composition of flavanones varied among varieties in our study. In most varieties, flavanones were mainly composed of hesperidin, narirutin, and vanillin, and hesperidin accounted for high proportions. However, for cocktail grapefruit and Changshanhuyou, naringin was the largest component, which is consistent with the finding from a previous study that naringin was the main flavonoid in grapefruit (21), followed by neohesperidin and neoeriocitrin in the peel, and neohesperidin and narirutin in the pulp. Our results were consistent with previous studies (11) that hesperidin, naringin, and neohesperidin were the main flavonoids in citrus peels, while Coelho et al. (25) also reported that flavonoids were the main polyphenols, with naringin and hesperidin dominating, followed by phenolic acids. The highest content of hesperidin was in the pulp of Himekoharu and the peel of Ponkan, which were similar to the contents of mandarin reported by Zhang et al. (28). Zhao et al. (19) concluded that hesperidin was the main flavonoid in tangerine but detected higher levels than ours. The difference might be attributed to multiple factors, such as variety, maturity, and origin environment (26).

Polymethoxylated flavones were a class of flavonoids in citrus that contained at least four methoxy groups and had beneficial physiological functions for human beings, such as a strong oxidative capacity to inhibit the growth of cancer cells *in vivo/in vitro* (29, 30). Nobiletin, tangerine, and sinensetin were the predominant PMFs in Chinese wild mandarin citrus, and they were also detected in clementine mandarin, satsume mandarin, navel orange, and common orange (30, 31). In our research, they were mainly found in citrus peels, but in the pulp, they were at insignificant levels. Our results were similar to Celano et al.'s study (32), which found that PMFs were mainly concentrated in citrus peels and varied considerably between varieties. Moreover, Ponkan, American tangerine, Mantouhong, Aishaju, and Himekohar were abundant with PMFs, and these varieties were the sources of PMFs.

Phenolic acids were the second-most abundant polyphenols in citrus, which were mainly composed of hydroxycinnamic acids and hydroxybenzoic acids, and their composition varied with variety and tissue parts. As in previous studies (20, 33), we observed that hydroxycinnamic acids were predominant and that ferulic acid was the main phenolic acid for all citrus varieties. Mantouhong and American tangerine were abundant in various phenolic acids except for vanillic acid. The peel of Ponkan contained the highest content of caffeic acid, and its *p*-coumaric acid and ferulic acid also were at high levels. In contrast, the abundance of all phenolic acids was poor in cocktail grapefruit and Changshanhuyou. Interestingly, Chen et al. (11) investigated the phenolic acids in the peels of 52 citrus varieties and found that benzoic acid and protocatechuic acid were the main phenolic acids; the difference might be caused by cultivar characteristics and environmental conditions.

Carotenoids were a generic term for C40 terpenoid compounds and their derivatives, which were widely found in plants. One of the most important appearance characteristics that influence consumer purchasing decisions is their accumulation in the peel. The color of the peel and pulp of ripe citrus fruit mainly depends on the content and composition of carotenoids and apocarotenoid pigments. Meanwhile, carotenoids in citrus were the main dietary source and were beneficial to human health, especially for the prevention of eye diseases (34, 35). Multari et al. (36) comprehensively analyzed phenolic compounds and carotenoids in tissues of four citrus varieties cultivated in southern Italy during their ripening stages, which delivered valuable information for utilization. Carotenoids also vary with citrus varieties, and genotype influences carotenoid accumulation (37, 38). In our study, β-cryptoxanthin was the main carotenoid in most varieties, and its content correlated with the color of the citrus fruit. Kato et al. (39) reported that the carotenoids detected in citrus pulp were mainly β-cryptoxanthin and zeaxanthin. Multari et al. (36) also reported that exocarp tissue was the main accumulator of carotenoids in citrus, while orange juice capsules were relatively low in carotenoids. Zeaxanthin was found to be highly detectable in the peel of some species, and Aishaju had the highest content of zeaxanthin. Besides, lutein was present at low levels in most varieties, but the peel of Ponkan had the highest lutein content.

Limonoids were highly oxygenated bioactive triterpenes and unique secondary metabolites to citrus and other plants of the family Rutaceae, which largely determined the quality of the fruit and showed great potential as a therapeutic agent for human health (40, 41). Liu et al. (18) revealed the structural diversity of limonoids and their tissue distribution in grapefruit fruit. The composition of limonoids in this study was dominated by limonin in both the pulp and peel, with American tangerine having the highest limonin content and Ponkan containing the highest nomilin content.

These secondary metabolites were genetically controlled and tied to the genetic background (11). We found that flavanones were strongly and positively correlated with each other, and positive correlations existed among PMFs, phenolic acids, carotenoids, and limonoids. Coelho et al.'s research (25) showed that concentrations of many phenolics varied greatly among the citrus samples, and they also suggested considering various phenolic components as possible parameters for the classification of citrus. However, the complexity of the morphology of the genus Citrus and the tendency to hybridize between genera complicated citrus classification. PCA and HCA were utilized to identify phenolic compounds and citrus classification (11, 25). PCA and HCA results indicated that metabolites of closely related varieties were similar and converged into the same group. Eventually, these varieties were divided into four groups by pulp and three groups by peel, and this led to the new notion that, in addition to PCA and HCA of the peel component, the pulp component could also be taken into account in citrus classification.

## 5. Conclusion

In this study, functional components including flavonoids, phenolic acids, carotenoids, and limonoids were fully analyzed in both pulp and peel from 11 citrus varieties, which were local hybrids and traditional varieties in Zhejiang, to explore the distribution pattern and variability between varieties. Secondary metabolites were far more abundant in the peel than in the pulp, and the accumulation varied significantly between species. Most of these components were correlated with each other. PCA and HCA results showed that citrus varieties were grouped according to these metabolites while also taking citrus peel and pulp into consideration. To the best of our knowledge, this study was the first to provide information on the citrus classification based on four secondary metabolites, and it included citrus peel and pulp. The obtained results filled the data gap for secondary metabolites from local citrus, which could provide data references for citrus resource utilization, selection, and breeding of superior varieties and other research varieties in the future perspective.

## Data availability statement

The original contributions presented in the study are included in the article/Supplementary material, further inquiries can be directed to the corresponding author.

## Author contributions

ML: conceptualization, methodology, software, formal analysis, resources, writing—original draft, visualization, project administration, and funding acquisition. CX: methodology, writing—original draft, formal analysis, and supervision. XG: formal analysis, software, and writing—review and editing. WZ: methodology and writing—review and editing. ZY: software and data curation. TW: supervision, validation, and investigation. XF: validation and writing—review and editing. YW: formal analysis, validation, and investigation. All authors contributed to the article and approved the submitted version.
