# Supplementary material for: Comparative study on secondary metabolites from different citrus varieties in the production area of Zhejiang
Source: Front Nutr. 2023 May 11;10:1159676. doi: 10.3389/fnut.2023.1159676 (PMC10211264; doi:10.3389/fnut.2023.1159676)
Supplement: Supplementary file 1 [file Data_Sheet_1.docx]

**Comparative study on secondary metabolites from different citrus varieties in the production area of Zhejiang**

Mei Lin^1,^*^,†^, Chengnan Xu^1,†^, Xueying Gao^2,3^, Weiqing Zhang^1^, Zhoulin Yao^1^, Tianyu Wang^1^, Xianju Feng^1^, Yue Wang^1^

^1^Zhejiang Citrus Research Institute, Taizhou, Zhejiang 318026, China

^2^Center for reproductive medicine, Ren Ji Hospital, School of Medicine, Shanghai Jiao Tong University, Shanghai 200120, China

^3^Shanghai Key Laboratory for Assisted Reproduction and Reproductive Genetics, Shanghai 200120, China

*Corresponding author. *E-mail addresses:* hylm84712002@126.com

^†^Equal contribution and first authorship: These authors contributed equally to this work and share first authorship.

**Table S1.** Information about 11 citrus varieties.

| **Varieties** | **Parents** | **Origin** | **Suitable harvest time** | **Category** | **Sampling time** | **Photographs of fruits** |
| --- | --- | --- | --- | --- | --- | --- |
| Aishaju | (*C. unshiu* × *C. clementina*) × (((*C. unshiu* × *C. sinensis*) × *C. unshiu*) × ((*C. erythrosa*×*C. paradisi*)× *C. reticulata*))× C. *reticulata* | China | Early December | Hybrid | 05/12/2021 | 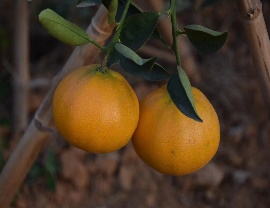 |
| Mantouhong | *Citrus reticulate* Blanco | China | Mid November | Mandarin | 20/11/2021 | 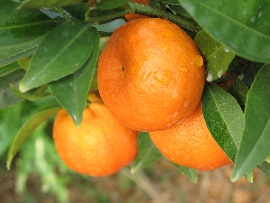 |
| American tangerine | *Citrus reticulate* Blanco | Japan | Late November | Mandarin | 30/11/2021 | 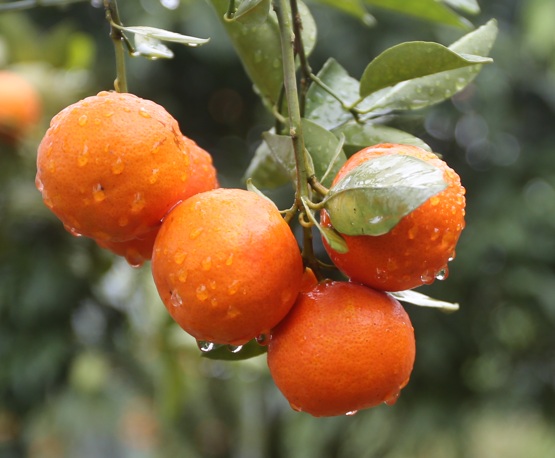 |
| Changshanhuyou | *Citrus paradisi* Macf. | China | Early December | Hybrid | 05/12/2021 | 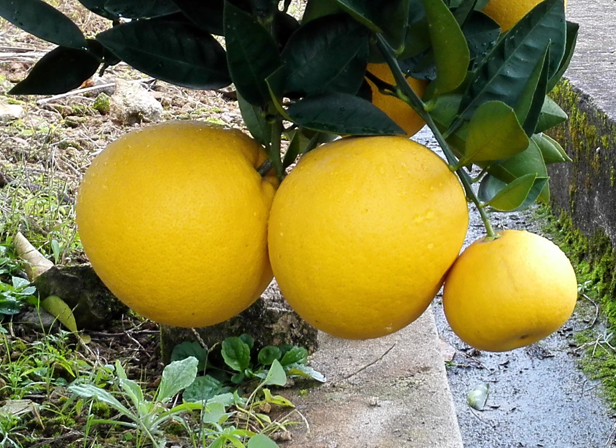 |

Continuation of Table S1.

| **Varieties** | **Parents** | **Origin** | **Suitable harvest time** | **Category** | **Sampling time** | **Photographs of fruits** |
| --- | --- | --- | --- | --- | --- | --- |
| Haruka | *Citrus tamurana* Hort. Ex Tanaka | Japan | Early December to Late February | Hybrid | 30/11/2021 | 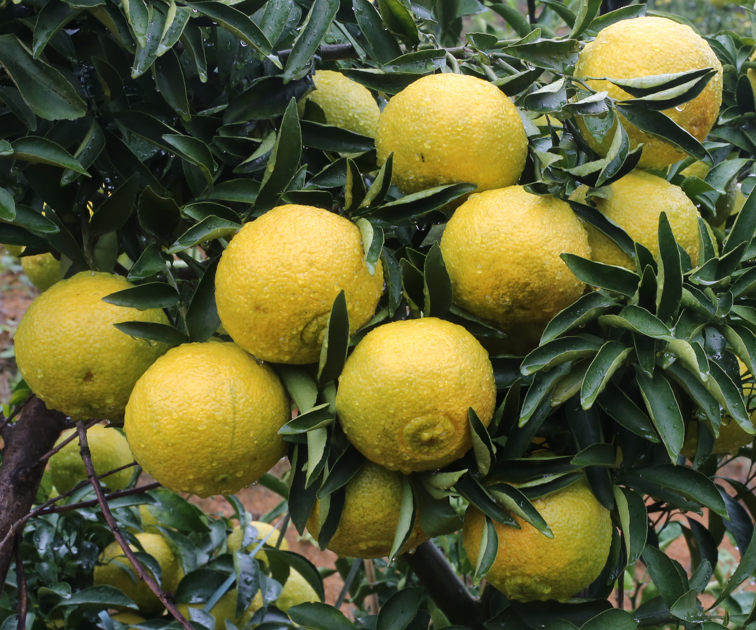 |
| Cocktail Grapefruit | *Citrus paradisi* Macf. | American | Early December | Hybrid | 05/12/2021 | 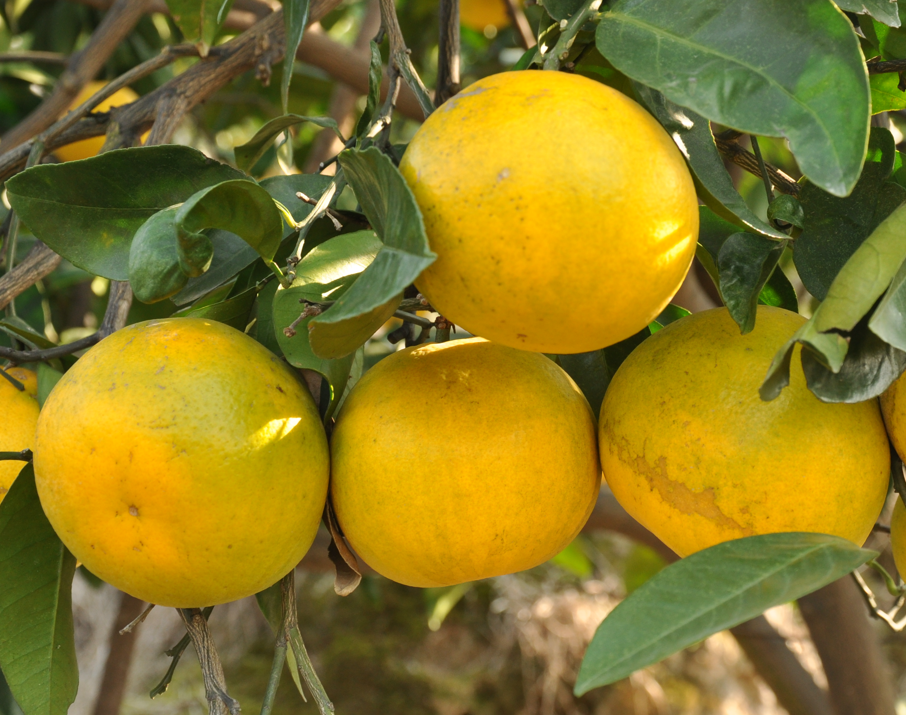 |
| Tian tangelo | *C. unshiu × (C. grandis × C. tangerine）* | Japan | Late November to Early December | Hybrid | 05/12/2021 | 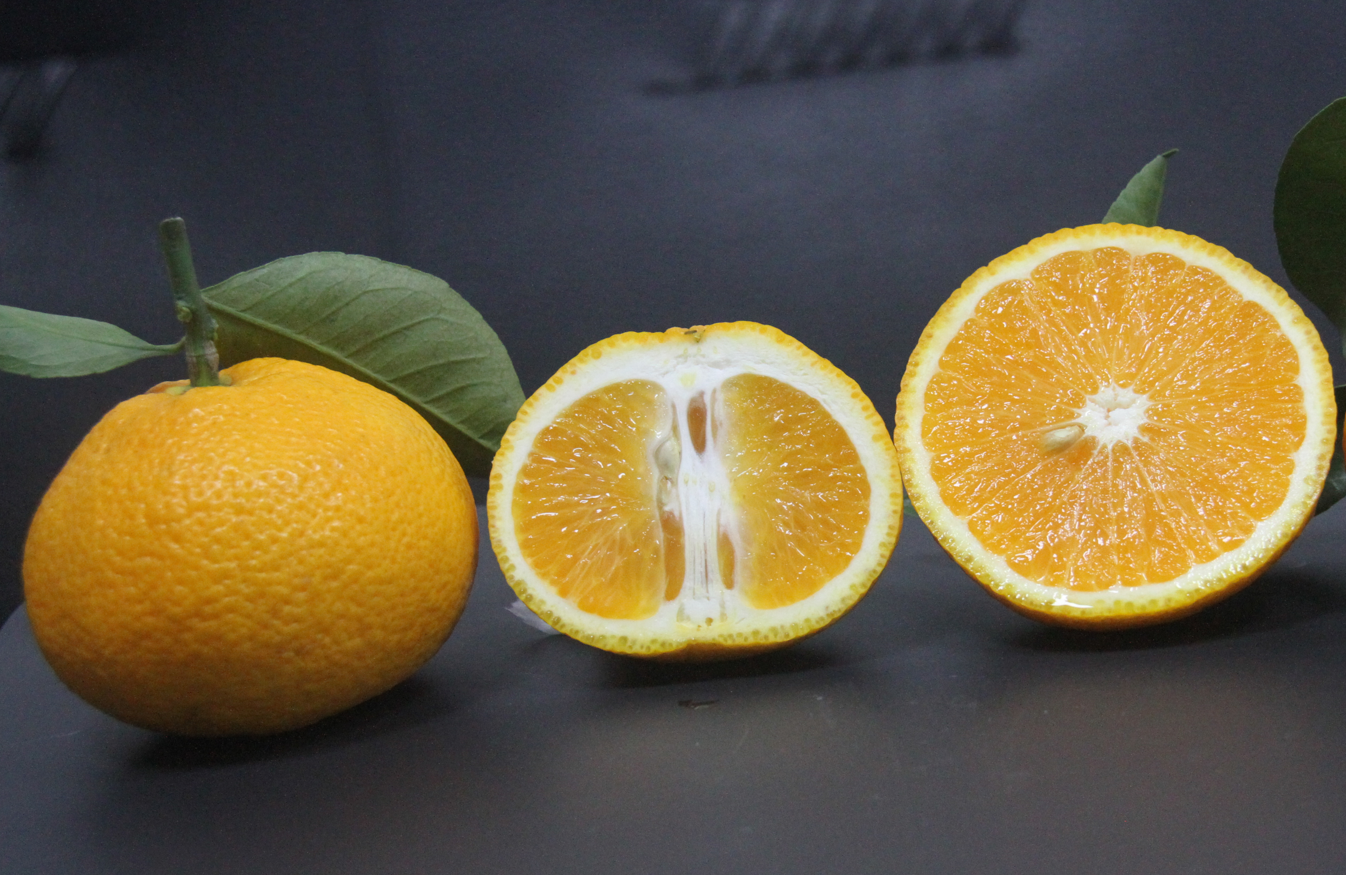 |
| Himekoharu | *((C. unshiu × C. sinensis) × C. sp.* | Japan | Late January to Late Februry | Hybrid | 30/01/2022 | 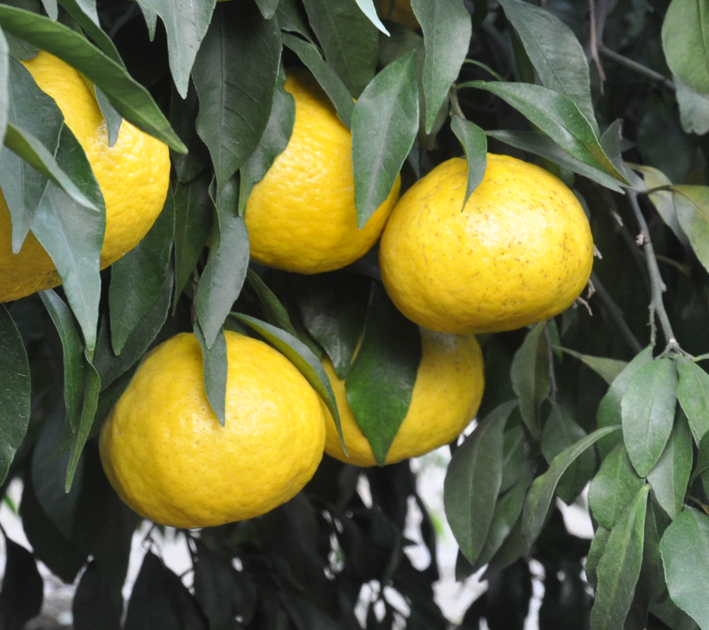 |

Continuation of Table S1.

| **Varieties** | **Parents** | **Origin** | **Suitable harvest time** | **Category** | **Sampling time** | **Photographs of fruits** |
| --- | --- | --- | --- | --- | --- | --- |
| Hongmeiren | *(C. unshiu × C. clementina) × (((C. unshiu × C. sinensis) × C. unshiu) × ((C. erythrosa×C. paradisi )× C. reticulata))* | Japan | Late November to Late January | Hybrid | 05/12/2021 | 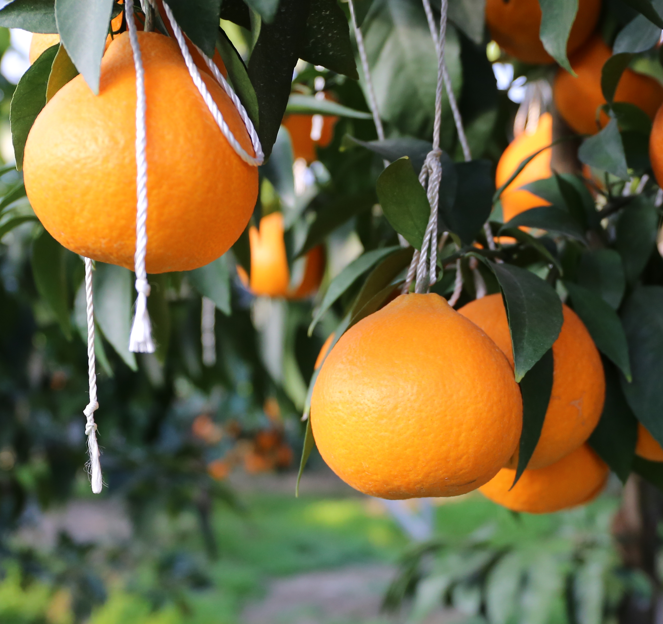 |
| Yura | *Citrus unshiu* Marc. | Japan | Mid October to Early November | Mandarin | 30/10/2021 | 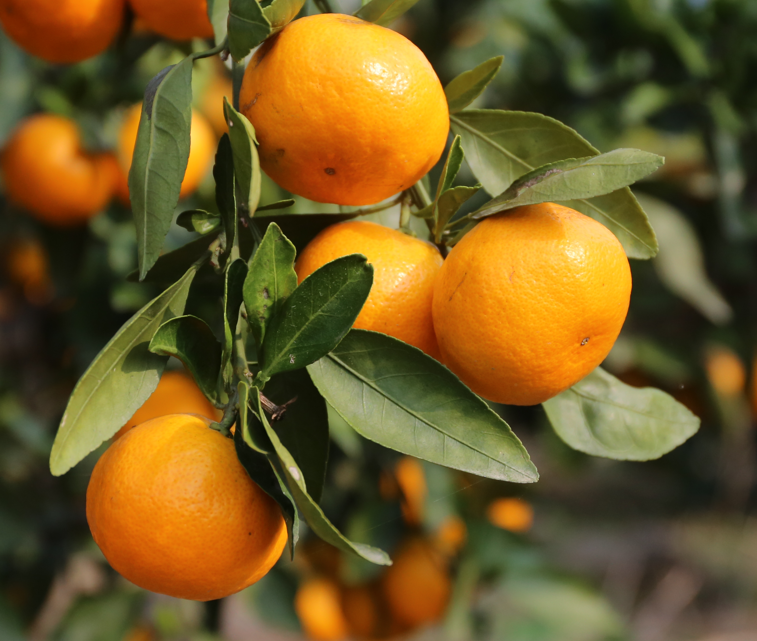 |
| Ponkan | *Citrus reticulata* Blanco | China | Late November to Early December | Mandarin | 05/12/2021 | 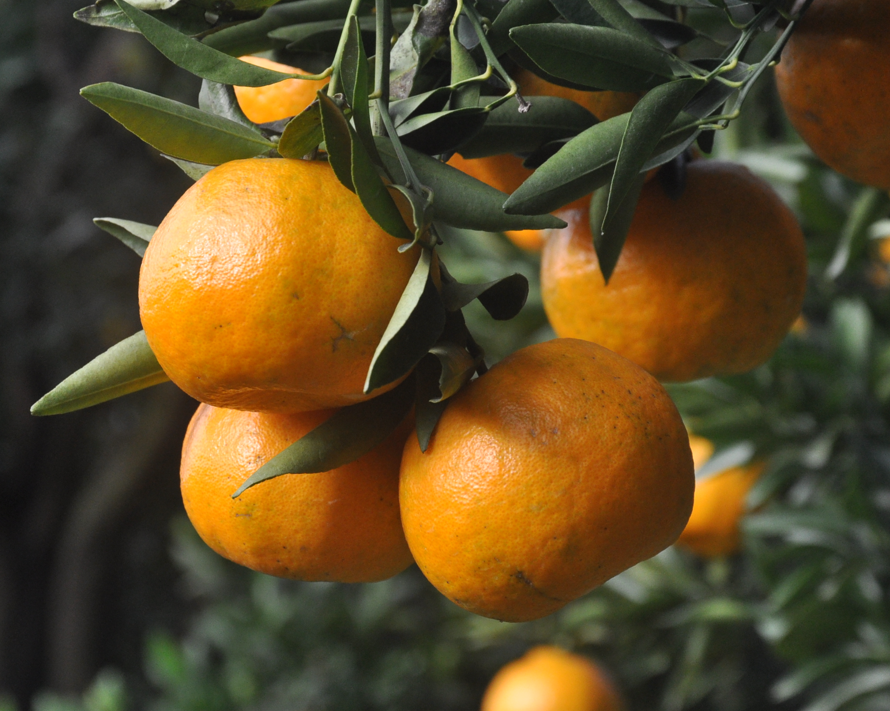 |

**Table S2.** Calibration curves of the standards.

| **Standards** | **Retention time**  **(s)** | **Detection wavelength**  **(nm)** | **Linear range**  **(mg/L)** | **Calibration curve** | **LOQ**  **(mg/L)** | ***R^2^*** |
| --- | --- | --- | --- | --- | --- | --- |
| Eriocitrin (1) | 6.624 | 283 | 0.02 ~ 200 | *y*=9917.85*x*-365.75 | 0.02 | 1.0000 |
| Neoeriocitrin (2) | 6.826 | 283 | 0.02 ~ 200 | *y*=11614.85*x*-4513.06 | 0.02 | 0.9998 |
| Narirutin (3) | 7.244 | 283 | 0.02 ~ 200 | *y*=8628.91*x*-2870.18 | 0.02 | 0.9999 |
| Naringin (4) | 7.396 | 283 | 0.02 ~ 200 | *y*=9432.96*x*-4566.62 | 0.02 | 0.9997 |
| Hesperidin (5) | 7.529 | 283 | 0.02 ~ 200 | *y*=10153.39*x*-4544.66 | 0.02 | 0.9998 |
| Neohesperidin (6) | 7.644 | 283 | 0.02 ~ 200 | *y*=10439.23*x*-4296.97 | 0.02 | 0.9998 |
| Vanillin (7) | 7.698 | 283 | 0.02 ~ 200 | *y*=5020.49*x*+153.33 | 0.02 | 1.0000 |
| Poncirin (8) | 8.490 | 283 | 0.02 ~ 200 | *y*=9064.52*x*-4434.47 | 0.02 | 0.9997 |
| Naringenin (9) | 8.561 | 283 | 0.02 ~ 200 | *y*=7784.58*x-*2476.23 | 0.02 | 0.9999 |
| Hesperetin (10) | 8.637 | 283 | 0.01 ~ 200 | *y*=18500.27*x*-3704.75 | 0.01 | 0.9999 |
| Rhoifolin (11) | 8.828 | 283 | 0.01 ~ 200 | *y*=20964.93*x*-3935.63 | 0.01 | 0.9999 |
| Apigenin (12) | 9.183 | 283 | 0.01 ~ 200 | *y*=25707.44*x*+3781.94 | 0.01 | 1.0000 |
| Sinensetin (13) | 9.605 | 330 | 0.01 ~ 200 | *y*=24275.4*x*-3804.08 | 0.01 | 0.9999 |
| Nobiletin (14) | 10.115 | 330 | 0.01 ~ 200 | *y*=20638.01*x*+195.81 | 0.01 | 1.0000 |
| Tangeretin (15) | 10.579 | 330 | 0.01 ~ 200 | *y*=22966.03*x*+7661.23 | 0.01 | 0.9999 |

*y* and *x* represent the Peak areas and the concentration of standards.

Continuation of Table S2.

| **Standards** | **Retention time**  **(s)** | **Detection wavelength**  **(nm)** | **Linear range**  **(mg/L)** | **Calibration curve** | **LOQ**  **(mg/L)** | ***R^2^*** |
| --- | --- | --- | --- | --- | --- | --- |
| Protocatechuic acid (16) | 2.329 | 220 | 0.02 ~ 200 | *y*=14853.90*x*-6671.55 | 0.02 | 0.9999 |
| *p*hydroxybenzonic acid (17) | 3.523 | 220 | 0.02 ~ 200 | *y*=19568.88*x*-3791.16 | 0.02 | 1.0000 |
| Caffeic acid (18) | 4.459 | 320 | 0.02 ~ 200 | *y*=21055.82*x*-13122.23 | 0.02 | 0.9997 |
| Vanillic acid (19) | 4.592 | 220 | 0.02 ~ 200 | *y*=13759.81*x*-1657.48 | 0.02 | 1.0000 |
| *p*-coumaric acid (20) | 6.079 | 320 | 0.02 ~ 200 | *y*=22507.62*x*-3234.52 | 0.02 | 1.0000 |
| Ferulic Acid (21) | 7.048 | 320 | 0.02 ~ 200 | *y*=18657.95*x*+9434.76 | 0.02 | 0.9998 |
| Sinapic acid (22) | 7.460 | 320 | 0.02 ~ 200 | *y*=18705.25*x*-7791.96 | 0.02 | 0.9999 |
| Lutein (23) | 11.159 | 450 | 0.1 ~ 20 | *y*=38257.59*x*-1372.04 | 0.1 | 1.0000 |
| Zeaxanthin (24) | 12.132 | 450 | 0.1 ~ 20 | *y*=37466.56*x*-3087.87 | 0.1 | 0.9999 |
| β-cryptoxanthin (25) | 15.165 | 450 | 0.1 ~ 20 | *y*=42910.24*x-*3447.58 | 0.1 | 0.9999 |
| α-carotene (26) | 17.597 | 450 | 0.1 ~ 20 | *y*=30323.49*x*-2576.13 | 0.1 | 0.9999 |
| β-carotene (27) | 19.775 | 450 | 0.1 ~ 20 | *y*=50850.75*x*-2394.42 | 0.1 | 1.0000 |
| Limonin (28) | 3.375 | 210 | 0.2 ~ 200 | *y*=3071.17*x*+922.63 | 0.2 | 1.0000 |
| Nomilin (29) | 4.598 | 210 | 0.2 ~ 200 | *y*=2724.22*x*+1004.20 | 0.2 | 1.0000 |

*y* and *x* represent the Peak areas and the concentration of standards.

**Table S3.** Correlation of secondary metabolites.

**Table S4.** Principal component scores of citrus varieties (4 principal components).

| Tissues  Varieties | pulp | | | | | | peel | | | | | |
| --- | --- | --- | --- | --- | --- | --- | --- | --- | --- | --- | --- | --- |
|  | F1 | F2 | F3 | F4 | F | Ranking | F1 | F2 | F3 | F4 | F | Ranking |
| Aishaju | 9.33 | 4.87 | 4.71 | -2.45 | 6.00 | 1 | 8.72 | 0.35 | 3.00 | -0.07 | 4.97 | 4 |
| Mantouhong | 8.32 | -1.52 | 1.41 | 2.76 | 3.99 | 4 | 9.68 | 1.61 | 1.27 | -1.51 | 5.39 | 3 |
| American tangerine | 8.34 | -3.09 | 2.92 | 3.56 | 4.06 | 3 | 11.15 | 2.54 | 3.11 | -2.62 | 6.49 | 2 |
| Changshanhuyou | -15.13 | 1.60 | 1.48 | 0.29 | -6.05 | 10 | -17.50 | 7.01 | 2.09 | 3.09 | -6.76 | 10 |
| Haruka | -1.20 | -4.61 | -5.79 | -1.63 | -3.00 | 8 | -4.74 | -5.08 | -2.22 | -1.47 | -4.10 | 9 |
| Cocktail Grapefruit | -17.77 | 1.94 | 4.79 | 1.38 | -6.33 | 11 | -15.48 | 5.48 | -2.46 | -3.32 | -7.37 | 11 |
| Tian tangelo | -4.11 | -2.05 | -2.47 | -3.23 | -3.20 | 9 | -3.54 | -4.25 | -1.92 | -1.07 | -3.20 | 8 |
| Himekoharu | 0.78 | 1.21 | -5.37 | 2.70 | -0.21 | 6 | -1.73 | -4.03 | 1.59 | 2.88 | -1.33 | 6 |
| Hongmeiren | -0.13 | -2.87 | -5.01 | -0.04 | -1.78 | 7 | -2.33 | -5.76 | -0.30 | 0.69 | -2.54 | 7 |
| Yura | 6.42 | 10.37 | -2.68 | -0.81 | 4.55 | 2 | -0.27 | -5.23 | 1.40 | 0.70 | -1.12 | 5 |
| Ponkan | 5.15 | -5.87 | 6.01 | -2.52 | 1.97 | 5 | 16.04 | 4.85 | -5.58 | 2.69 | 8.98 | 1 |
